# Degradation of fish food webs in the Anthropocene

**DOI:** 10.1126/sciadv.adu6540

**Published:** 2026-02-18

**Authors:** Juan D. Carvajal-Quintero, Maria Dornelas, Lise Comte, Juliana Herrera-Pérez, Pablo A. Tedesco, Xingli Giam, Ulrich Brose, Jonathan M. Chase

**Affiliations:** ^1^ German Centre for Integrative Biodiversity Research (iDiv) Halle-Jena-Leipzig, Leipzig, Germany.; ^2^ Department of Biology, Dalhousie University, Halifax, NS B3H 4R2, Canada.; ^3^ Centre for Biological Diversity, School of Biology, University of St. Andrews, St. Andrews, UK.; ^4^ MARE—Centro de Ciências do Mar e do Ambiente, Faculdade de Ciências, Universidade de Lisboa, Lisbon, Portugal.; ^5^ Conservation Science Partners, Inc., Truckee, CA, USA.; ^6^ Laboratorio de Macroecología Evolutiva, Red de Biología Evolutiva, Instituto de Ecología, Veracruz, México.; ^7^ Centre de Recherche sur la Biodiversité et l’Environnement (CRBE), Université de Toulouse, CNRS, IRD, Toulouse INP, Université Toulouse 3–Paul Sabatier (UT3), Toulouse, France.; ^8^ Department of Ecology and Evolutionary Biology, The University of Tennessee, Knoxville, TN, USA.; ^9^ Institute of Biodiversity, Friedrich Schiller University, Jena, Germany.; ^10^ Institute for Computer Science, Martin Luther University Halle-Wittenberg, Halle 06108, Germany.

## Abstract

Global change reshapes biodiversity through shifts in species composition, richness, and body size. How these shifts combine to alter higher-level ecological processes within food webs can have important implications for entire ecosystems. However, the strength and direction of these shifts will depend on combinations of ways that species and trait compositions change through time. We combine long-term data from ~15,000 freshwater and marine fish communities (1949–2019) with information about their size, diets, and trophic status to evaluate how food webs change through time at local spatial scale. We found that selective species turnover driven by body size reductions is associated with widespread alteration to fish food web topology and function, including increased connectance and generalism, leading to higher predation pressure and increased prey vulnerability. Food webs were also less modular. These changes extend across food web trophic structures, causing a cascading shift in the proportion of species across trophic levels. Our study highlights complex biodiversity responses to confluent changes across multiple facets.

## Teaser

The decrease in body size driven by the selective species turnover is widely altering fish food web topology and function.

## INTRODUCTION

Anthropogenic activities have extensively affected nature and the biodiversity within, leading to rapid changes in the numbers and types of species in local assemblages (*1*–*4*). These changes often favor species with certain traits and disfavor others (*5*–*7*), which can in turn alter patterns of ecosystem functioning and related ecological processes (*8*–*10*). For example, anthropogenic activities often disproportionately alter the body size of species within populations and assemblages (*6*, *11*, *12*), and these changes can transform the structure and function of food webs. Body size is a critical trait that shapes feeding interactions and the overall architecture of food webs (*13*–*17*). A well-known principle in most food webs is that predators are consistently larger than their prey (*13*, *18*–*20*). This body size ratio determines the trophic niche of predators (*13*, *16*, *17*, *20*), i.e., the feeding preferences of predators and the range of prey sizes they can effectively consume. Consequently, any change in species composition that affects the body size distribution of predators or preys within a community can alter the predator-to-prey size ratio and lead to changes in the structure and functioning of food webs. Although widespread and substantial shifts in species composition and body size have been documented among communities through space and time (*1*, *6*, *21*, *22*), whether these shifts lead to consistent, predictable effects on food web structure and functioning remains a major, unresolved question.

**Fig. 1.**
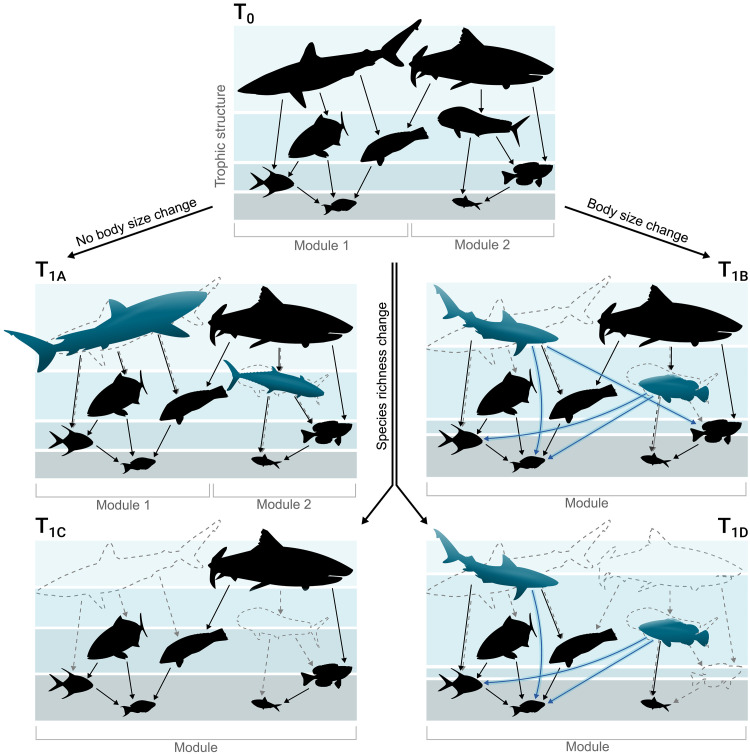
Pathways of temporal changes in taxonomic diversity and adult body size resulting in different potential scenarios of local food web changes. Species turnover (represented by species dashed and blue silhouettes) unfolds from T_0_ (top) to T_1_ (below), reshaping species composition and potentially affecting species richness, body size, and species interaction. Changes in species composition not affecting body size are depicted on the left (T_1A_ and T_1C_), while those altering body size are on the right (T_1B_ and T_1D_). Black solid lines represent interactions among species within the food webs, blue lines indicate interaction pathways that emerged following species turnover, and dashed lines show interactions that were lost due to species turnover. When a black line appears alongside a dashed line, it denotes interaction pathways present in the original food web (T_0_) that are now fulfilled by a new species (blue silhouettes). When turnover does not affect species richness nor body size, no structural changes are expected in the food webs (T_1A_). However, alterations in body size arrangement can change the prey selectivity of the species (blue lines) inducing food web shifts even in the absence of changes in species richness (T_1B_). When species turnover alters species richness, it directly affects food web topology by changing the number of interacting species and links (T_1C_). Moreover, if turnover reduces both species richness and body size, cumulative effects on food web structure and functioning are anticipated (T_1D_). Ultimately, changes in species composition that affect species richness and body size can alter the distribution of species across different trophic groups (as indicated by the variation in the width of the bands behind the food webs). These changes may also result in a decrease in modularity within the networks (T_1B_, T_1C_, and T_1D_), depicted at the bottom of each figure panel by horizontal brackets.

Changes in species richness directly affect the number of nodes (species) and links (interactions) in a food web (*16*). Likewise, changes in species body size alter the predator-to-prey size ratio and the distribution of links across nodes in a food web (*14*, *16*). The cumulative effects of species turnover and body size changes within communities can therefore alter the structure of food webs via several alternative pathways (Fig. 1). First, there can be no change in local species richness at time 0 (Fig. 1, T_0_), as is often observed (*1*, *2*, *23*). If there is no change in species richness and also no change in important traits like body size (Fig. 1, scenario T_1A_), we would expect no change in food web structure. This is because, even if species composition changes, the number of species and their body size distribution remain unchanged. As a result, the number of nodes and the trophic niche of predators within the food web stay stable. However, even if there are no changes in species richness, the average body size of species in the community can decrease if there are changes in the identity of the species in a community through time (Fig. 1, scenario T_1B_) (*6*). Here, we would expect a shift in the structure and function of the food web because the trophic niche of a species is often determined by its body size. Therefore, the selective filtering of body sizes can introduce new trophic interactions, rewiring food webs by reshaping the density and distribution of links, and consequently altering their structure and functioning (*13*, *14*, *17*, *24*). If species richness changes through time, another set of possibilities emerges. For brevity, we consider scenarios where species richness declines without (Fig. 1, scenario T_1C_) or with (Fig. 1, scenario T_1D_) concomitant changes in body size, but species richness increases would simply be the inverse. If species richness declines, but body size does not change (Fig. 1, scenario T_1C_), we expect a change in the structure of the food web via changes in the number of nodes in the food web and consequently a less connected network (lower connectance) due to the decrease in the number of interactions (*25*, *26*). Alternatively, if both species richness (number of nodes) and body size change (Fig. 1, scenario T_1D_), we would expect a simultaneous reduction in the network size and connectance along with the food web rewiring caused by the changes of predator-prey interactions resulting from alterations in species’ body sizes (*13*, *14*, *17*, *24*). Ultimately, changes in species composition that affect species richness, body size, or both (T_1B_, T_1C_, and T_1D_) can also alter the distribution of species across different trophic groups. This may occur either through the selective loss from a particular trophic level (TL) (*27*, *28*) or through shifts in the composition of species’ body sizes, given that body size generally increases with predator TL (*29*, *30*). These changes in the trophic structure may also result in a decrease in modularity within the networks—the presence of distinct clusters of interactions—which is crucial for maintaining stability within ecological networks (*31*–*33*).

Here, we present a comprehensive assessment of how the alteration of species composition and adult body size within local fish assemblages has reshaped the topology and functioning of their food webs over the past decades. To do so, we compiled an extensive database of fish assemblage time series from freshwater and marine ecosystems [RivFishTIME and BioTIME; (*34*, *35*)]. These data encompass a diverse array of 15,029 fish assemblages containing 2844 fish species and were sourced from assemblages surveyed in 103 studies from across the world (fig. S1). Time series ranged from 2 to 70 years. After standardizing sampling effort across time series (*1*, *36*), we assessed temporal trends in species richness, dissimilarity in species composition, and body size (measured as maximum species size). We then linked these shifts in the taxonomic diversity and species traits to changes in food web metrics at topological (e.g., network connectance and modularity) and functional level (e.g., trophic similarity and prey vulnerability) associated with food web stability and resilience (*31*–*33*). These metrics were estimated from food webs reconstructed using a predator-prey model calibrated with a comprehensive database of over 23,000 trophic interactions and body size records for co-occurring fish species pairs (see Methods). To explore temporal trends in these metrics, we used hierarchical generalized linear models, nesting spatial-unit time series within their original studies to address potential biases arising from the nonindependence of spatial-unit time series within a given study (see Methods). We fitted each biodiversity metric (i.e., taxonomic diversity, body size, and food web metrics) separately and reported the sensitivity of the rate of changes (model slope) to the replicate variability. We considered any 95% confidence interval (CI) not overlapping zero as compelling evidence for a directional trend (see Methods).

## RESULTS AND DISCUSSION

### Trends in local assemblages: Species richness, composition, and body size

**Fig. 2.**
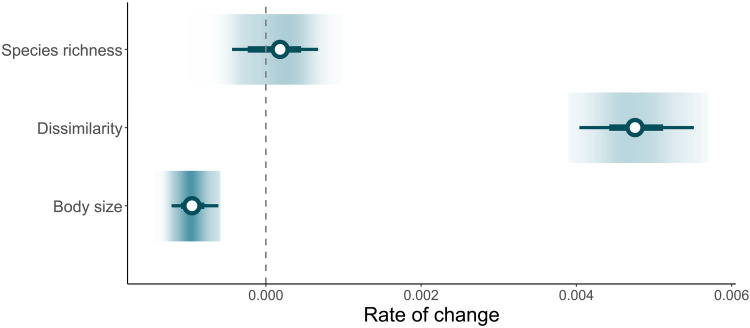
Changes in assemblage metrics per year. The gradient plots display the distribution of slopes for changes in species richness, dissimilarity in species composition, and body size. Darker colors correspond to higher densities. The horizontal bars with error bars denote the mean and the 50% and 95% CIs of the mean estimates (depicted by white circles).

We found no overall trend in species richness through time (mean: 0.0001 per year; 95% CI: −0.0003, 0.0006; Fig. 2), but a strong directional change in dissimilarity in species composition (mean: 0.0047; 95% CI: 0.0040, 0.0055; Fig. 2), an increasingly well-known phenomenon across taxa (*1*, *2*, *21*, *23*). We found that these compositional changes were nonrandom, with selective filtering toward smaller species reflecting an overall downsizing of body size in species assemblages over time (mean: −0.0009; 95% CI: −0.0012, −0.0006; Fig. 2) (see pathway T_1B_ in Fig. 1 for illustration). This downsizing of fish communities has been observed elsewhere (*6*) and is frequently associated with human-driven overexploitation, warming, and reduced resource availability (*11*, *37*). However, owing to the pervasive importance of body size (*14*, *38*), changes in assemblage-level body sizes are likely to alter the structure of food webs and the ecosystems in which these changes take place.

### Topological and functional changes in food webs

**Fig. 3.**
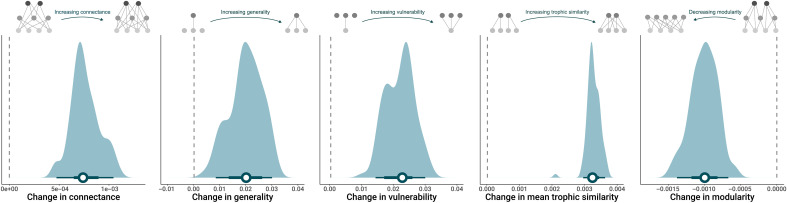
Changes in food web topology metrics and trophic similarity per year. The density plots display the distribution of slopes for changes in connectance, generality, vulnerability (predation pressure), trophic similarity, and modularity. The horizontal bars with error bars denote the mean and the 50% and 95% CIs of the mean estimates (depicted by white circles). The top insets illustrate the changes in the number and distribution of links that each trend represents, with the arrow representing the direction of the change from T_0_ (aligned with the dotted line representing no change) to T_1_.

Over time, we found that the connectance of fish food webs increased (mean: 0.0007; 95% CI: 0.0004, 0.0010; Fig. 3). This was because there was an increase in mean network generality (mean: 0.0199; 95% CI: 0.0083, 0.0300; Fig. 3), indicating that temporal turnover favors more generalist species that feed on a broader range of prey. Such replacement of specialists by generalists is a frequently observed signature of perturbed environments (*5*), and thus, we expect increases in network connectance to be a general phenomenon. We also observed an overall decrease in food web modularity through time (mean: −0.0010; 95% CI: −0.0014, −0.0007; Fig. 3), which describes the degree of species frequently interacting in clusters. This decline in modularity can be attributed to two processes. First, modularity can decline through time because of increases in generalist species (fig. S2), which disrupt the typical block-like structure of ecological networks by interacting broadly with a diverse prey array (*39*, *40*). Second, modularity can decline because of a reduction in the proportion of top predators (fig. S2) that anchor modules in ecological networks by interacting with various species at lower TLs, forming distinct subgroups of interactions (*14*, *27*, *41*). These topological changes may have important implications for the stability and the maintenance of biodiversity, since species interaction networks largely influence the response of communities and ecosystems to environmental change (*42*, *43*). For example, the increased connectance and reduced modularity can destabilize ecosystems by synchronizing the responses of food webs to perturbations (*39*). Reductions in the degree of modularity can also compromise the stability of food webs because it fosters frequent interactions within clusters that previously limited the propagation of species-specific impacts across the food web (*31*, *32*, *44*).

At the functional level, we found increases in mean network generality (mean: 0.0199; 95% CI: 0.0083, 0.0300; Fig. 3), trophic similarity (mean: 0.0032; 95% CI: 0.0029, 0.0035; Fig. 3), and prey vulnerability (mean: 0.0217; 95% CI: 0.0137, 0.0295; Fig. 3). These results indicate that temporal turnover tends to favor generalist species that have wider trophic habits. Because the number of species remains stable over time, the increase of generalists increases the density of links in the food web, causing a combined increase in diet overlap (i.e., increased trophic similarity) and predation pressure (i.e., higher prey vulnerability), as preys are targeted by more predators. Generalist predators tend to be more adaptable to changing environmental conditions, enabling them to colonize assemblages (*5*, *39*, *45*, *46*). While generalist species can enhance network robustness by providing alternative interaction pathways (*47*), an excessive dominance of generalists can have the opposite effect. By increasing trophic similarity, it can lead to prey overexploitation and the competitive exclusion of specialists (*48*), ultimately making food webs more vulnerable to environmental disturbances and triggering cascades of extinctions (*39*, *48*, *49*).

**Fig. 4.**
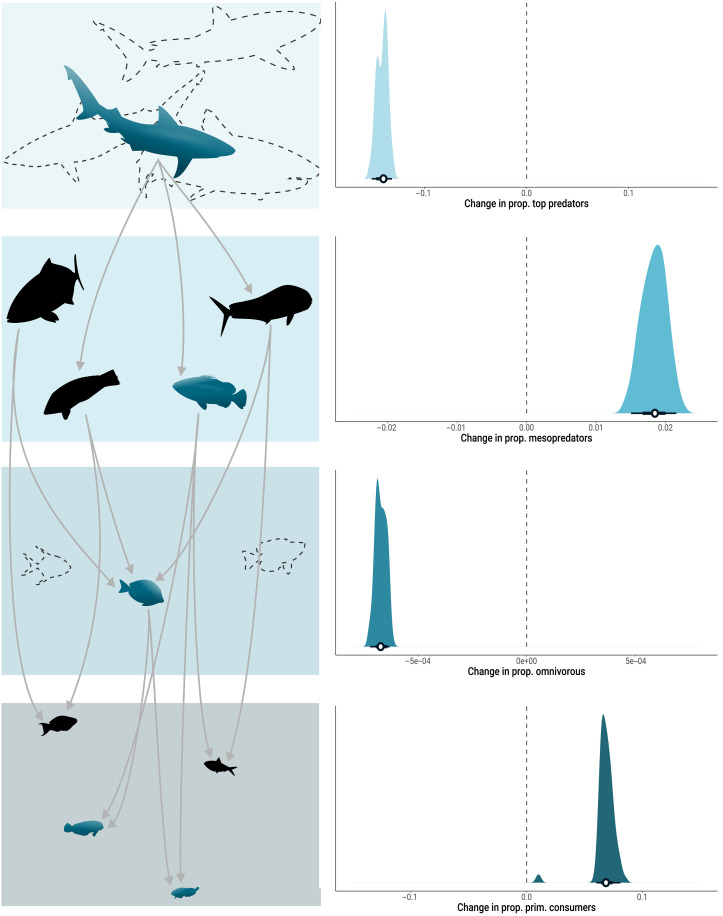
Changes in proportion of trophic groups per year. The density plots (right) display the distribution of slopes for changes in proportion within the assemblages of top predators, mesopredators, omnivorous, and primary consumers. The horizontal bars with error bars denote the mean and the 50% and 95% CIs of the mean estimates (depicted by white circles). On the left are illustrated schematic changes in the proportion of species across trophic groups to help with the interpretation. Dashed silhouettes indicate species lost, blue silhouettes represent new species, and black silhouettes denote species present across the entire food web time series. Changes in species composition result from both species loss and the arrival of new ones, reshaping the trophic structure of the food web. These shifts reduce the number of top predators and omnivores while increasing the number of mesopredators and primary consumers.

Across all time series, we found cascading shifts among adjacent groups in the trophic hierarchy (see pathway T_1B_ in Fig. 1 for illustration). Specifically, the proportion of species of top predators and omnivores within the food web declined, while mesopredators and primary consumers increased through time (Fig. 4). This pattern of opposite trends among adjacent trophic groups emphasizes the interconnected nature of the trophic hierarchy in food webs and the potential repercussions of altering one level on the entire food web. These contrasting trends among adjacent trophic groups also suggest that the loss of apex predators is disrupting top-down ecological forces within food webs and altering the biotic filters that shape species assemblages. This results in a release of mesopredators (*27*, *50*, *51*), which triggers local turnover among lower trophic-level species and reshapes trophic structure. While trophic structures have tended to be stable for hundreds to many thousands of years amid substantial shifts in species composition (*52*, *53*), our observation of widespread transformation in the trophic structure of aquatic food webs underscores the severity of Anthropocene impacts on biodiversity.

We found no consistent differences when we repeated our analysis with time series spanning different time periods (figs. S3 to S5), minimum number of species (figs. S6 to S8), and sampling coverage (figs. S9 to S11), suggesting that these findings are robust to time series length, network size, and species sampling detectability. In addition, the directions of the effects we observed in food web structural changes were generally similar across both freshwater and marine environments, despite differences in trophic similarity and the proportion of omnivorous species (figs. S12 and S13). These generally consistent effects across environments suggest that the degradation of food webs is widespread across ecosystems. Nevertheless, we recognize the limitations stemming from the geographic biases of the data used in our study. For example, like most such syntheses, our data are notably biased toward Europe, North America, and Australasia (fig. S1). The challenge of accurately quantifying biodiversity trends is heightened by the scarcity of long-term biodiversity data in many parts of the world, particularly in tropical regions [e.g., (*54*)]. Thus, our estimated mean trends are not suitable for geographical extrapolation, but rather can only serve as a representation of the most current state of knowledge derived from existing open data (*55*, *56*). In addition, the topology-based metrics we used assume that all species and interactions in the network are equally important (*57*). However, changes in species abundance—a typical response to global change (*58*)—can substantially affect the functioning of food webs by altering species dominance and the strength of interactions in food webs. Future studies could complement our approach using node- and link-weighted metrics to more deeply understand how community restructuring in the Anthropocene affects food webs, as well as the implications of these changes for ecosystem functioning and human well-being (*57*). Last, our theoretical framework (Fig. 1) is grounded in the allometric rule where predator feeding niches are defined in relation to body size (*17*, *59*). This approach assumes no variation in feeding specialization within predator size classes. While body size remains a key determinant of predator specialization, with larger species typically being more generalist (*14*, *17*, *59*, *60*), intraspecific differences in foraging strategies can introduce additional complexity that warrants deeper investigation.

In summary, we show that selective turnover of species via reductions in body size is degrading the topology and trophic structure of fish food webs in the Anthropocene. Notably, our findings emphasize that, despite the absence of a general trend in species richness change over time (*1*, *2*, *21*, *23*), a noticeable transformation in the functioning of biodiversity within food webs is occurring through the filtering of previously unknown trait arrangements (an illustration of this process is shown in Fig. 1, pathway T_1B_). Food webs are becoming structurally less complex due to the loss of modularity, while becoming more generalized with increases in connectance, diet breadth, and overlap. This degradation of food webs is a widespread pattern across thousands of fish communities spanning diverse regions and environments. Theory suggests that if these trends persist, they could critically undermine food web stability and resilience. The concurrent rise in generalization and decline in structural complexity and trophic diversity may reduce the capacity of food webs to buffer against perturbations that can propagate and increase vulnerability to shared environmental pressures (*31*, *32*, *44*, *48*). While we can only speculate on the causes of these fish food web changes across many different systems experiencing an array of different human threats, we suspect that they are tied to the historical and ongoing degradation of fish fauna. Factors such as widespread and targeted exploitation of marine and freshwater biota, habitat destruction, and climate shifts have resulted in regional and local extinctions and population declines of some species, particularly larger predatory fish (*61*, *62*). Fish are crucial for supporting aquatic biodiversity, economies, livelihoods, nutritional security, and cultural practices on our planet (*63*), and as such, understanding the magnitude and timing of food web changes resulting from strong human-induced disruption is essential for their effective conservation and sustainable management (*33*, *64*). Our results highlight the importance of monitoring species interaction networks to better understand the functional effects of biodiversity change in the Anthropocene.

## METHODS

Our analysis integrates ecological assemblage, trait data (body size), and documented trophic interactions to reconstruct the time series of fish food webs and elucidate the topological and functional changes occurring within them. We drew upon two existing sources of published ecological assemblages, extracting time series information for both freshwater and marine environments. Species’ body sizes and trophic interactions were sourced from a combination of published literature and open-access databases. Below, we outline the standardization process and the calculations used for reconstructing the time series of food webs. In addition, we present the statistical analyses detailing the trends observed in fish food webs during the Anthropocene era (see fig. S14).

To ensure consistency in the merging of the different datasets and databases, we harmonized taxonomy across the databases following the methodology outlined in (*65*), using FishBase (*66*) as a taxonomic reference to rectify synonyms and correct misspellings for fish using the “rfishbase” package (*67*).

### Data collection and selection

#### Assemblage time series

We obtained ecological assemblage data from the BioTIME (*35*) and RivFishTIME (*34*) databases, which are the most extensive global, open-access repositories of assemblage composition time series. BioTIME encompasses studies on various taxonomic groups, whereas RivFishTIME specifically focuses on freshwater fish. We gathered the studies on both freshwater and marine fish from BioTIME and integrated them with those in RivFishTIME. Each study encompasses distinct samples surveyed consistently over time using a standardized methodology, in which the number of years sampled can change.

Because we aimed to quantify biodiversity changes at a local scale, we adopted established methods (*36*) to identify and standardize studies that had large extents and multiple sampling locations, by partitioning them into spatial units. For marine environments, we used a global grid comprised of 96-km^2^ hexagonal cells as spatial unit [using dggridR; (*68*)], whereas for freshwater environments, we used a global layer of subbasins spanning, on average, 99 km^2^ [based on HydroBASIN level 12; (*69*)]. Studies that were contained within a single spatial unit (i.e., grid cell or subbasin) remained unpartitioned. Subsequently, each sample received a unique combination of study ID and spatial unit based on its latitude and longitude. This assignment generated a distinctive identifier for each assemblage time series within spatial units, ensuring the preservation of both study and sample integrity.

To minimize the impact of unobserved species on our biodiversity change estimates, we computed the abundance-based coverage (*70*) for each (annual) sample within each spatial unit–level time series, excluding all the time series without records of abundance. We removed all samples with coverage less than 0.85 based on the ratio of detected to expected species richness (*36*).

Last, we implemented a sample-based rarefaction to standardize the number of samples across years within each time series (*36*, *71*). In this process, we prioritized surveys conducted in the same climatic season, distinguishing between the warm season (from April to September) and the cold season (from October to March) in the Northern Hemisphere, and vice versa for the Southern Hemisphere. The rarefaction was repeated 100 times for each time series, and the outcomes of each iteration were preserved to reconstruct the food webs.

#### Body size

We gathered data on body size traits, specifically the maximum reported body length, from both FishBase and published literature (*72*). This value was treated as a fixed species-level trait and applied uniformly to all records of that species across time. We used this information to evaluate the trends in species body size within the assemblages and to model the probability of interaction between species (see below).

#### Species diets

To compile species diet data from scientific literature, we gathered information from various sources. We collected diet data from FishBase (*66*) and the Global Biotic Interaction database [GloBI; (*73*)]. In addition, we curated an independent dataset of fish diet records by consolidating information from journal articles and natural history handbooks (*72*). For journal articles, we conducted Google Scholar queries using the scientific names of each fish species to identify relevant studies and acquired records not included in FishBase and GloBI. In each diet record, we documented consumer-resource interactions at the species level for the consumer and, when possible, at the highest taxonomic level for the resource. In cases where taxonomic information was unavailable for the resources, we retained the common names (*72*). In addition, when data were available, we noted the life stage at which the interaction occurred and retained only those interactions that occurred at the adult stage. The diet information was subsequently used to calculate the TLs of the species and to calibrate the niche model used to reconstruct the food webs (see below).

#### Trophic groups

Species were grouped into distinct trophic groups based on their TLs using predefined thresholds from FishBase (*66*). The trophic groups include top predators (TL > 4), mesopredators (TL = 2.8 to 4), omnivores (TL = 2.2 to 2.79), and primary consumers (TL = 2 to 2.19). The calculation of TL values was performed using the “dietr” package (*74*). This methodology calculates fractional TLs based on both quantitative and qualitative data on diet items, mirroring the approach used in estimating species’ TLs within the FishBase database.

### Reconstruction of food web time series

To reconstruct the food webs for each fish community at each time step, we modeled the probability that a focal species preys on a candidate species with which it co-occurs, applying a trait-matching function based on the niche model for food web structure (*59*), where the log body size of the predator determines its optimum and the range of its trophic niche, while log prey size determines its position in the predator niche (*17*). We used a Gaussian function to describe the probability that a predator species *i* feeds on a potential prey species *j* (*P_ij_*)

$$P_{\mathrm{ij}}=\theta_{i}\;\text{exp}\;\left(-\frac{{\left(m_{i}-m_{j}-\mu_{i}\right)}^{2}}{2\sigma_{i}^{2}}\right)$$

where $m_{i}$ and $m_{j}$ are the log_10_ body sizes of predator *i* and prey *j*, $\mu_{i}$ is the log_10_ optimal size ratio of predator *i*, and $\sigma_{i}^{2}$ defines the feeding range of predator *i*. The parameter $\theta_{i}$ represents the maximum feeding probability of predator *i*.

Thus, with available data on observed predator-prey interactions and their body size, the model can be calibrated to define a predation window defining the body size range within which a predator species of a specific size can feed (*17*, *75*). Ultimately, the model indicates whether the focal consumer species preys on a candidate resource species.

To calibrate and assess the performance of the model, we used a data subset of predator-prey interactions derived from the data compilation on species diets (see the “Species diets” section in Methods). This data subset encompasses 23,834 predator-prey interactions at the species level, involving 1396 predator species and 2515 prey species. These interactions cover diverse locations in both freshwater and marine environments, capturing a broad spectrum of environmental conditions (*72*).

To select the model used in the reconstruction of the food webs, we evaluated multiple models with different windows of predation (quantiles 0.01 to 0.99, 0.02 to 0.98, 0.03 to 0.97, 0.04 to 0.96, and 0.05 to 0.95) in different fish groups (marine and freshwater). For each model, we split the predator-prey dataset into two parts: 70% of the dataset was randomly chosen to calibrate the model, whereas the remaining 30% was used to evaluate it. We calculated the Boyce index to evaluate the quality of the models and select those with the highest values (marine fish: 0.05 to 0.95, mean Boyce index = 0.56, SD = 0.09, *n* = 999; freshwater fish: 0.03 to 0.97, mean Boyce index = 0.54, SD = 0.08, *n* = 999) (fig. S15). The Boyce index varies from −1 to +1, where a positive value indicates a model whose predictions are consistent with the presence of interactions in the evaluation dataset, values close to zero mean that the model is not different from a random interaction, while values close to −1 indicate an incorrect model, which predicts unsuitable interactions (*76*, *77*). A Boyce index of ≥0.5 indicates a well-performing model, where predictions match the actual distribution of observed interactions (*77*). This cross-validation procedure was repeated 999 times. After identifying the most performant models, we applied them separately to the 2090 marine and 822 freshwater species present in the assemblage time series using their respective body size data (refer to the “Body size” section in Methods). This enabled us to identify all potential predator-prey interactions that may occur among all the species in the database and subsequently allocate them only to the specific pair of species (predator-prey) that co-occur in a particular sampling. Because of the size-based nature of our model, it can misidentify trophic interactions forbidden in nature (e.g., large herbivorous fish feeding on smaller fish). To correct this potential issue, we removed all predator interactions for fishes whose documented diet was not classified as piscivorous.

Modeling predator-prey interactions based on species traits is a common approach for predicting interactions within food webs across different scenarios of species composition. This includes the reconstruction of historical food webs based on past species composition and the generation of current food webs in regions where direct recordings are lacking [e.g., (*16*, *49*, *78*, *79*)]. Besides, this method helps to overcome sampling uncertainty inherent in traditional methods, by identifying likely interactions that may not have been detected and filling gaps in undersampled ecological networks. Ecologists typically have a limited understanding of food webs, as even intensive sampling efforts often fall short in fully capturing interactions (*80*, *81*). The difficulty lies in detecting interactions, which are often infrequent or occur between rare species (*80*–*82*). Recognizing this, there has been a growing effort over the past decade to construct models that forecast unobserved links within ecological networks (*83*, *84*). Our study builds on these previous efforts, aiming to capture all potential interactions among the set of co-occurring fish species even if not yet documented in the literature, driven by the imperative to better understand ecological networks and their transformations resulting from human-induced effects in biodiversity.

Although trait-based inferences can be advantageous in reconstructing predator-prey interactions, relying solely on traits also comes with some limitations. This is because a singular trait value per species overlooks intraspecific trait variation, such as potential variations between populations of the same species in different environments (*85*), which could potentially lead to an underestimation of niche breadth and species interactions (*86*). The selection of trait and interaction records from global databases, particularly for rare or poorly studied species, could also represent another crucial source of uncertainty and bias, as these records are more likely to capture out-of-the-range observations, ultimately affecting model predictions [e.g., (*87*)].

### Dataset description

For the analysis presented here, the database comprises 100 replicates (or iterations) of 15,029 food web time series reconstructed for rarefied fish assemblages data from 103 different studies or monitoring programs, including 2844 species. Specifically, 12,467 of these time series focus on marine environments, while 2562 focus on freshwater environments. To ensure a sufficiently large network size for metric measurement, all time series adhere to a minimum rule of having at least five species across the sampling period. Geographically, the data exhibit a notable skew toward Europe, North America, and Australasia, as illustrated in fig. S1. The time series in the database span a mean duration of 16.84 (±10.75) years, with a corresponding mean of 6.31 (±5.56) sampling years. For accessibility, all utilized data are available in the code repository listed in the Supplementary Materials (*72*).

### Calculation of taxonomic diversity, body size, and food web metrics

Within each time series, we calculated several metrics describing different biodiversity facets.

#### Taxonomic diversity

1) Temporal dissimilarity in species composition: Evaluated through pairwise Jaccard dissimilarity between the first year and subsequent years in the time series.

2) Species richness: Determined as the number of species/nodes in a given year in a time series.

#### Body size

The mean body size is the average body size of species in a given year in a time series.

#### Food webs

Within the food webs, we calculated different metrics related with the network topology, species’ role, and food web functioning.

1) Topological: (i) Connectance: The proportion of realized interactions out of all possible bipartite interactions, expressed as the ratio between the number of links and the square of the number of species. (ii) Modularity: Describes the presence of aggregated sets of interacting species, indicating the extent to which interactions occur more frequently within modules than between modules.

2) Species’02 role: (i) Generality: Represents the mean number of prey taxa consumed per predator. (ii) Prey vulnerability: Indicates the mean number of predators consuming each prey taxon. (iii) Mean trophic similarity: Calculated as the average trophic similarities between all species in a food web. Trophic similarity between two species is defined by the number of common prey and predators divided by their total number of preys and predators.

3) Functioning: Trophic group proportions: The proportion of species within a specific trophic group (i.e., top predators, mesopredators, omnivorous, and primary consumers) relative to the total number of species within the food web.

### Statistical analysis: Models of trends

We used mixed-effects models to examine temporal variations in all biodiversity metrics, encompassing taxonomic diversity, body size, and food web metrics. In the fixed model structure, the selected biodiversity metric served as the response variable, with year (mean-centered) as the sole fixed independent variable. In addition, year was included as a random slope, varying across studies and spatial units, which represent the smallest reported sampling units. To address the nonindependence of the time series, spatial units were nested within the original studies from which they were derived. We used Poisson, normal, and binomial error structures to model biodiversity changes based on the distribution of the response variable (i.e., biodiversity metric). All statistical models were implemented within a frequentist framework using the “glmmTMB” package (*85*) in R [v4.2.1; (*88*)]. The overarching model structure in “glmmTMB” annotation is as follows:

# gaussian family function: glmmTMB(*biodiversity metric* ~ *Year_Cent* + (*Year_Cent*|*Study_ID_All*/*Spatial_Uni_ID*), data = data_Mod_TL_BS2, family = gaussian); # poisson family function: glmmTMB(*biodiversity metric* ~ *Year_Cent* + (*Year_Cent*|*Study_ID_All*/*Spatial_Uni_ID*), data = data_Mod_TL_BS2, family = poisson); # binomial family function: glmmTMB(cbind(*Number of species in a trophic level*, *Total species in the assemblage*) ~ *Year_Cent* + (*Year_Cent*|*Study_ID_All*/*Spatial_Uni_ID*), data = data_Mod_TL_BS2, family = binomial)

Each biodiversity metric’s model was fitted to the 100 iterations (*89*) of the time series datasets to account for potential variations in species composition resulting from standardizing sample effort using sample-based rarefaction (see the “Assemblage time series” section in Methods). Subsequently, we extracted the slope from each model fit and combined them to draw inferences by comparing the mean coefficient and the CIs across 100 iterations, accounting for the uncertainty in the assemblage time series.

## References

[1] S. A. Blowes, S. R. Supp, L. H. Antão, A. Bates, H. Bruelheide, J. M. Chase, F. Moyes, A. Magurran, B. McGill, I. H. Myers-Smith, M. Winter, A. D. Bjorkman, D. E. Bowler, J. E. K. Byrnes, A. Gonzalez, J. Hines, F. Isbell, H. P. Jones, L. M. Navarro, P. L. Thompson, M. Vellend, C. Waldock, M. Dornelas, The geography of biodiversity change in marine and terrestrial assemblages. Science 366, 339–345 (2019). 31624208 10.1126/science.aaw1620

[2] M. Dornelas, N. J. Gotelli, B. McGill, H. Shimadzu, F. Moyes, C. Sievers, A. E. Magurran, Assemblage time series reveal biodiversity change but not systematic loss. Science 344, 296–299 (2014). 24744374 10.1126/science.1248484

[3] M. Dornelas, N. J. Gotelli, H. Shimadzu, F. Moyes, A. E. Magurran, B. J. McGill, A balance of winners and losers in the Anthropocene. Ecol. Lett. 22, 847–854 (2019). 30874368 10.1111/ele.13242

[4] S. L. Lewis, M. A. Maslin, Defining the anthropocene. Nature 519, 171–180 (2015). 25762280 10.1038/nature14258

[5] J. Clavel, R. Julliard, V. Devictor, Worldwide decline of specialist species: Toward a global functional homogenization? Front. Ecol. Environ. 9, 222–228 (2011).

[6] I. S. Martins, F. Schrodt, S. A. Blowes, A. E. Bates, A. D. Bjorkman, V. Brambilla, J. Carvajal-Quintero, C. F. Y. Chow, G. N. Daskalova, K. Edwards, N. Eisenhauer, R. Field, A. Fontrodona-Eslava, J. J. Henn, R. van Klink, J. S. Madin, A. E. Magurran, M. McWilliam, F. Moyes, B. Pugh, A. Sagouis, I. Trindade-Santos, B. J. McGill, J. M. Chase, M. Dornelas, Widespread shifts in body size within populations and assemblages. Science 381, 1067–1071 (2023). 37676959 10.1126/science.adg6006

[7] P. S. Stewart, A. Voskamp, L. Santini, M. F. Biber, A. J. M. Devenish, C. Hof, S. G. Willis, J. A. Tobias, Global impacts of climate change on avian functional diversity. Ecol. Lett. 25, 673–685 (2022). 35199917 10.1111/ele.13830

[8] M. W. Cadotte, Functional traits explain ecosystem function through opposing mechanisms. Ecol. Lett. 20, 989–996 (2017). 28639274 10.1111/ele.12796

[9] M. McLean, A. Auber, N. A. J. Graham, P. Houk, S. Villéger, C. Violle, W. Thuiller, S. K. Wilson, D. Mouillot, Trait structure and redundancy determine sensitivity to disturbance in marine fish communities. Glob. Change Biol. 25, 3424–3437 (2019). 10.1111/gcb.14662 31006156

[10] D. A. Wardle, R. D. Bardgett, R. M. Callaway, W. H. Van der Putten, Terrestrial ecosystem responses to species gains and losses. Science 332, 1273–1277 (2011). 21659595 10.1126/science.1197479

[11] M. Daufresne, K. Lengfellner, U. Sommer, Global warming benefits the small in aquatic ecosystems. Proc. Natl. Acad. Sci. U.S.A. 106, 12788–12793 (2009). 19620720 10.1073/pnas.0902080106 PMC2722360

[12] F. A. Smith, R. E. Elliott Smith, S. K. Lyons, J. L. Payne, Body size downgrading of mammals over the late quaternary. Science 360, 310–313 (2018). 29674591 10.1126/science.aao5987

[13] U. Brose, P. Archambault, A. D. Barnes, L.-F. Bersier, T. Boy, J. Canning-Clode, E. Conti, M. Dias, C. Digel, A. Dissanayake, A. A. V. Flores, K. Fussmann, B. Gauzens, C. Gray, J. Häussler, M. R. Hirt, U. Jacob, M. Jochum, S. Kéfi, O. McLaughlin, M. M. MacPherson, E. Latz, K. Layer-Dobra, P. Legagneux, Y. Li, C. Madeira, N. D. Martinez, V. Mendonça, C. Mulder, S. A. Navarrete, E. J. O’Gorman, D. Ott, J. Paula, D. Perkins, D. Piechnik, I. Pokrovsky, D. Raffaelli, B. C. Rall, B. Rosenbaum, R. Ryser, A. Silva, E. H. Sohlström, N. Sokolova, M. S. A. Thompson, R. M. Thompson, F. Vermandele, C. Vinagre, S. Wang, J. M. Wefer, R. J. Williams, E. Wieters, G. Woodward, A. C. Iles, Predator traits determine food-web architecture across ecosystems. Nat. Ecol. Evol. 3, 919–927 (2019). 31110252 10.1038/s41559-019-0899-x

[14] G. Woodward, B. Ebenman, M. Emmerson, J. M. Montoya, J. M. Olesen, A. Valido, P. H. Warren, Body size in ecological networks. Trends Ecol. Evol. 20, 402–409 (2005). 16701403 10.1016/j.tree.2005.04.005

[15] K. L. Wootton, A. Curtsdotter, T. Roslin, R. Bommarco, T. Jonsson, Towards a modular theory of trophic interactions. Funct. Ecol. 37, 26–43 (2023).

[16] E. C. Fricke, C. Hsieh, O. Middleton, D. Gorczynski, C. D. Cappello, O. Sanisidro, J. Rowan, J.-C. Svenning, L. Beaudrot, Collapse of terrestrial mammal food webs since the Late Pleistocene. Science 377, 1008–1011 (2022). 36007038 10.1126/science.abn4012

[17] D. Gravel, T. Poisot, C. Albouy, L. Velez, D. Mouillot, Inferring food web structure from predator–prey body size relationships. Methods Ecol. Evol. 4, 1083–1090 (2013).

[18] U. Brose, T. Jonsson, E. L. Berlow, P. Warren, C. Banasek-Richter, L.-F. Bersier, J. L. Blanchard, T. Brey, S. R. Carpenter, M.-F. C. Blandenier, L. Cushing, H. A. Dawah, T. Dell, F. Edwards, S. Harper-Smith, U. Jacob, M. E. Ledger, N. D. Martinez, J. Memmott, K. Mintenbeck, J. K. Pinnegar, B. C. Rall, T. S. Rayner, D. C. Reuman, L. Ruess, W. Ulrich, R. J. Williams, G. Woodward, J. E. Cohen, Consumer–resource body-size relationships in natural food webs. Ecology 87, 2411–2417 (2006). 17089649 10.1890/0012-9658(2006)87[2411:cbrinf]2.0.co;2

[19] C. Barnes, D. Maxwell, D. C. Reuman, S. Jennings, Global patterns in predator–prey size relationships reveal size dependency of trophic transfer efficiency. Ecology 91, 222–232 (2010). 20380211 10.1890/08-2061.1

[20] J. Li, M. Luo, S. Wang, B. Gauzens, M. R. Hirt, B. Rosenbaum, U. Brose, A size-constrained feeding-niche model distinguishes predation patterns between aquatic and terrestrial food webs. Ecol. Lett. 26, 76–86 (2023). 36331162 10.1111/ele.14134

[21] M. Dornelas, J. M. Chase, N. J. Gotelli, A. E. Magurran, B. J. McGill, L. H. Antão, S. A. Blowes, G. N. Daskalova, B. Leung, I. S. Martins, F. Moyes, I. H. Myers-Smith, C. D. Thomas, M. Vellend, Looking back on biodiversity change: Lessons for the road ahead. Philos. Trans. R. Soc. B. Biol. Sci. 378, 20220199 (2023). 10.1098/rstb.2022.0199 PMC10225864 37246380

[22] J. C. D. Terry, J. D. O’Sullivan, A. G. Rossberg, No pervasive relationship between species size and local abundance trends. Nat. Ecol. Evol. 6, 140–144 (2022). 34969990 10.1038/s41559-021-01624-8 PMC8825279

[23] M. Vellend, L. Baeten, I. H. Myers-Smith, S. C. Elmendorf, R. Beauséjour, C. D. Brown, P. De Frenne, K. Verheyen, S. Wipf, Global meta-analysis reveals no net change in local-scale plant biodiversity over time. Proc. Natl. Acad. Sci. U.S.A. 110, 19456–19459 (2013). 24167259 10.1073/pnas.1312779110 PMC3845118

[24] J. D. Yeakel, J. W. Moore, P. R. Guimarães, M. A. M. de Aguiar, Synchronisation and stability in river metapopulation networks. Ecol. Lett. 17, 273–283 (2014). 24304967 10.1111/ele.12228

[25] A. Danet, M. Mouchet, W. Bonnaffé, E. Thébault, C. Fontaine, Species richness and food-web structure jointly drive community biomass and its temporal stability in fish communities. Ecol. Lett. 24, 2364–2377 (2021). 34423526 10.1111/ele.13857

[26] M. M. Pires, M. Benchimol, L. R. Cruz, C. A. Peres, Terrestrial food web complexity in Amazonian forests decays with habitat loss. Curr. Biol. 33, 389–396.e3 (2023). 36580916 10.1016/j.cub.2022.11.066

[27] J. A. Estes, J. Terborgh, J. S. Brashares, M. E. Power, J. Berger, W. J. Bond, S. R. Carpenter, T. E. Essington, R. D. Holt, J. B. C. Jackson, R. J. Marquis, L. Oksanen, T. Oksanen, R. T. Paine, E. K. Pikitch, W. J. Ripple, S. A. Sandin, M. Scheffer, T. W. Schoener, J. B. Shurin, A. R. E. Sinclair, M. E. Soulé, R. Virtanen, D. A. Wardle, Trophic downgrading of planet Earth. Science 333, 301–306 (2011). 21764740 10.1126/science.1205106

[28] D. Pauly, V. Christensen, J. Dalsgaard, R. Froese, F. Torres, Fishing down marine food webs. Science 279, 860–863 (1998). 9452385 10.1126/science.279.5352.860

[29] T. N. Romanuk, A. Hayward, J. A. Hutchings, Trophic level scales positively with body size in fishes. Glob. Ecol. Biogeogr. 20, 231–240 (2011).

[30] F. W. Keppeler, C. G. Montaña, K. O. Winemiller, The relationship between trophic level and body size in fishes depends on functional traits. Ecological monographs 90, e01415 (2020).

[31] J. Grilli, T. Rogers, S. Allesina, Modularity and stability in ecological communities. Nat. Commun. 7, 12031 (2016). 27337386 10.1038/ncomms12031 PMC4931019

[32] D. B. Stouffer, J. Bascompte, Understanding food-web persistence from local to global scales. Ecol. Lett. 13, 154–161 (2010). 19968697 10.1111/j.1461-0248.2009.01407.x

[33] J. M. Tylianakis, E. Laliberté, A. Nielsen, J. Bascompte, Conservation of species interaction networks. Biol. Conserv. 143, 2270–2279 (2010).

[34] L. Comte, J. Carvajal-Quintero, P. A. Tedesco, X. Giam, U. Brose, T. Erős, A. F. Filipe, M.-J. Fortin, K. Irving, C. Jacquet, S. Larsen, S. Sharma, A. Ruhi, F. G. Becker, L. Casatti, G. Castaldelli, R. B. Dala-Corte, S. R. Davenport, N. R. Franssen, E. García-Berthou, A. Gavioli, K. B. Gido, L. Jimenez-Segura, R. P. Leitão, B. McLarney, J. Meador, M. Milardi, D. B. Moffatt, T. V. T. Occhi, P. S. Pompeu, D. L. Propst, M. Pyron, G. N. Salvador, J. A. Stefferud, T. Sutela, C. Taylor, A. Terui, H. Urabe, T. Vehanen, J. R. S. Vitule, J. O. Zeni, J. D. Olden, RivFishTIME: A global database of fish time-series to study global change ecology in riverine systems. Glob. Ecol. Biogeogr. 30, 38–50 (2021).

[35] M. Dornelas, L. H. Antão, F. Moyes, A. E. Bates, A. E. Magurran, D. Adam, A. A. Akhmetzhanova, W. Appeltans, J. M. Arcos, H. Arnold, N. Ayyappan, G. Badihi, A. H. Baird, M. Barbosa, T. E. Barreto, C. Bässler, A. Bellgrove, J. Belmaker, L. Benedetti-Cecchi, B. J. Bett, A. D. Bjorkman, M. Błażewicz, S. A. Blowes, C. P. Bloch, T. C. Bonebrake, S. Boyd, M. Bradford, A. J. Brooks, J. H. Brown, H. Bruelheide, P. Budy, F. Carvalho, E. Castañeda-Moya, C. A. Chen, J. F. Chamblee, T. J. Chase, L. S. Collier, S. K. Collinge, R. Condit, E. J. Cooper, J. H. C. Cornelissen, U. Cotano, S. K. Crow, G. Damasceno, C. H. Davies, R. A. Davis, F. P. Day, S. Degraer, T. S. Doherty, T. E. Dunn, G. Durigan, J. E. Duffy, D. Edelist, G. J. Edgar, R. Elahi, S. C. Elmendorf, A. Enemar, S. K. M. Ernest, R. Escribano, M. Estiarte, B. S. Evans, T.-Y. Fan, F. T. Farah, L. L. Fernandes, F. Z. Farneda, A. Fidelis, R. Fitt, A. M. Fosaa, G. A. D. C. Franco, G. E. Frank, W. R. Fraser, H. García, R. C. Gatti, O. Givan, E. Gorgone-Barbosa, W. A. Gould, C. Gries, G. D. Grossman, J. R. Gutierréz, S. Hale, M. E. Harmon, J. Harte, G. Haskins, D. L. Henshaw, L. Hermanutz, P. Hidalgo, P. Higuchi, A. Hoey, G. V. Hoey, A. Hofgaard, K. Holeck, R. D. Hollister, R. Holmes, M. Hoogenboom, C. Hsieh, S. P. Hubbell, F. Huettmann, C. L. Huffard, A. H. Hurlbert, N. M. Ivanauskas, D. Janík, U. Jandt, A. Jażdżewska, T. Johannessen, J. Johnstone, J. Jones, F. A. M. Jones, J. Kang, T. Kartawijaya, E. C. Keeley, D. A. Kelt, R. Kinnear, K. Klanderud, H. Knutsen, C. C. Koenig, A. R. Kortz, K. Král, L. A. Kuhnz, C.-Y. Kuo, D. J. Kushner, C. Laguionie-Marchais, L. T. Lancaster, C. M. Lee, J. S. Lefcheck, E. Lévesque, D. Lightfoot, F. Lloret, J. D. Lloyd, A. López-Baucells, M. Louzao, J. S. Madin, B. Magnússon, S. Malamud, I. Matthews, K. P. McFarland, B. McGill, D. McKnight, W. O. McLarney, J. Meador, P. L. Meserve, D. J. Metcalfe, C. F. J. Meyer, A. Michelsen, N. Milchakova, T. Moens, E. Moland, J. Moore, C. M. Moreira, J. Müller, G. Murphy, I. H. Myers-Smith, R. W. Myster, A. Naumov, F. Neat, J. A. Nelson, M. P. Nelson, S. F. Newton, N. Norden, J. C. Oliver, E. M. Olsen, V. G. Onipchenko, K. Pabis, R. J. Pabst, A. Paquette, S. Pardede, D. M. Paterson, R. Pélissier, J. Peñuelas, A. Pérez-Matus, O. Pizarro, F. Pomati, E. Post, H. H. T. Prins, J. C. Priscu, P. Provoost, K. L. Prudic, E. Pulliainen, B. R. Ramesh, O. M. Ramos, A. Rassweiler, J. E. Rebelo, D. C. Reed, P. B. Reich, S. M. Remillard, A. J. Richardson, J. P. Richardson, I. van Rijn, R. Rocha, V. H. Rivera-Monroy, C. Rixen, K. P. Robinson, R. R. Rodrigues, D. de Cerqueira Rossa-Feres, L. Rudstam, H. Ruhl, C. S. Ruz, E. M. Sampaio, N. Rybicki, A. Rypel, S. Sal, B. Salgado, F. A. M. Santos, A. P. Savassi-Coutinho, S. Scanga, J. Schmidt, R. Schooley, F. Setiawan, K.-T. Shao, G. R. Shaver, S. Sherman, T. W. Sherry, J. Siciński, C. Sievers, A. C. da Silva, F. R. da Silva, F. L. Silveira, J. Slingsby, T. Smart, S. J. Snell, N. A. Soudzilovskaia, G. B. G. Souza, F. M. Souza, V. C. Souza, C. D. Stallings, R. Stanforth, E. H. Stanley, J. M. Sterza, M. Stevens, R. Stuart-Smith, Y. R. Suarez, S. Supp, J. Y. Tamashiro, S. Tarigan, G. P. Thiede, S. Thorn, A. Tolvanen, M. T. Z. Toniato, Ø. Totland, R. R. Twilley, G. Vaitkus, N. Valdivia, M. I. Vallejo, T. J. Valone, C. V. Colen, J. Vanaverbeke, F. Venturoli, H. M. Verheye, M. Vianna, R. P. Vieira, T. Vrška, C. Q. Vu, L. V. Vu, R. B. Waide, C. Waldock, D. Watts, S. Webb, T. Wesołowski, E. P. White, C. E. Widdicombe, D. Wilgers, R. Williams, S. B. Williams, M. Williamson, M. R. Willig, T. J. Willis, S. Wipf, K. D. Woods, E. J. Woehler, K. Zawada, M. L. Zettler, BioTIME: A database of biodiversity time series for the Anthropocene. Glob. Ecol. Biogeogr. 27, 760–786 (2018). 30147447 10.1111/geb.12729 PMC6099392

[36] L. H. Antão, A. E. Bates, S. A. Blowes, C. Waldock, S. R. Supp, A. E. Magurran, M. Dornelas, A. M. Schipper, Temperature-related biodiversity change across temperate marine and terrestrial systems. Nat. Ecol. Evol. 4, 927–933 (2020). 32367031 10.1038/s41559-020-1185-7

[37] N. E. Bosch, J. Monk, J. Goetze, S. Wilson, R. C. Babcock, N. Barrett, J. Clough, L. M. Currey-Randall, D. V. Fairclough, R. Fisher, B. A. Gibbons, D. Harasti, E. S. Harvey, M. R. Heupel, J. L. Hicks, T. H. Holmes, C. Huveneers, D. Ierodiaconou, A. Jordan, N. A. Knott, H. A. Malcolm, D. McLean, M. Meekan, S. J. Newman, B. Radford, M. J. Rees, B. J. Saunders, C. W. Speed, M. J. Travers, C. B. Wakefield, T. Wernberg, T. J. Langlois, Effects of human footprint and biophysical factors on the body-size structure of fished marine species. Conserv. Biol. 36, e13807 (2022). 34312893 10.1111/cobi.13807 PMC9292308

[38] B. J. Enquist, A. J. Abraham, M. B. J. Harfoot, Y. Malhi, C. E. Doughty, The megabiota are disproportionately important for biosphere functioning. Nat. Commun. 11, 699 (2020). 32019918 10.1038/s41467-020-14369-y PMC7000713

[39] T. J. Bartley, K. S. McCann, C. Bieg, K. Cazelles, M. Granados, M. M. Guzzo, A. S. MacDougall, T. D. Tunney, B. C. McMeans, Food web rewiring in a changing world. Nat. Ecol. Evol. 3, 345–354 (2019). 30742106 10.1038/s41559-018-0772-3

[40] E. C. Fricke, J.-C. Svenning, Accelerating homogenization of the global plant–frugivore meta-network. Nature 585, 74–78 (2020). 32879498 10.1038/s41586-020-2640-y

[41] W. J. Ripple, J. A. Estes, R. L. Beschta, C. C. Wilmers, E. G. Ritchie, M. Hebblewhite, J. Berger, B. Elmhagen, M. Letnic, M. P. Nelson, O. J. Schmitz, D. W. Smith, A. D. Wallach, A. J. Wirsing, Status and ecological effects of the world’s largest carnivores. Science 343, 1241484 (2014). 24408439 10.1126/science.1241484

[42] B. J. Cardinale, J. E. Duffy, A. Gonzalez, D. U. Hooper, C. Perrings, P. Venail, A. Narwani, G. M. Mace, D. Tilman, D. A. Wardle, A. P. Kinzig, G. C. Daily, M. Loreau, J. B. Grace, A. Larigauderie, D. S. Srivastava, S. Naeem, Biodiversity loss and its impact on humanity. Nature 486, 59–67 (2012). 22678280 10.1038/nature11148

[43] T. H. Oliver, M. S. Heard, N. J. B. Isaac, D. B. Roy, D. Procter, F. Eigenbrod, R. Freckleton, A. Hector, C. D. L. Orme, O. L. Petchey, V. Proença, D. Raffaelli, K. B. Suttle, G. M. Mace, B. Martín-López, B. A. Woodcock, J. M. Bullock, Biodiversity and resilience of ecosystem functions. Trends Ecol. Evol. 30, 673–684 (2015). 26437633 10.1016/j.tree.2015.08.009

[44] L. J. Gilarranz, B. Rayfield, G. Liñán-Cembrano, J. Bascompte, A. Gonzalez, Effects of network modularity on the spread of perturbation impact in experimental metapopulations. Science 357, 199–201 (2017). 28706071 10.1126/science.aal4122

[45] M. J. Vander Zanden, J. M. Casselman, J. B. Rasmussen, Stable isotope evidence for the food web consequences of species invasions in lakes. Nature 401, 464–467 (1999).

[46] R. Kassen, The experimental evolution of specialists, generalists, and the maintenance of diversity. J. Evol. Biol. 15, 173–190 (2002).

[47] J. A. Dunne, R. J. Williams, N. D. Martinez, Network structure and biodiversity loss in food webs: Robustness increases with connectance. Ecol. Lett. 5, 558–567 (2002).

[48] D. Gilljam, A. Curtsdotter, B. Ebenman, Adaptive rewiring aggravates the effects of species loss in ecosystems. Nat. Commun. 6, 8412 (2015). 26400367 10.1038/ncomms9412

[49] L. Pecuchet, M.-A. Blanchet, A. Frainer, B. Husson, L. L. Jørgensen, S. Kortsch, R. Primicerio, Novel feeding interactions amplify the impact of species redistribution on an Arctic food web. Glob. Change Biol. 26, 4894–4906 (2020). 10.1111/gcb.15196 32479687

[50] K. R. Crooks, M. E. Soulé, Mesopredator release and avifaunal extinctions in a fragmented system. Nature 400, 563–566 (1999).

[51] J. F. Bruno, M. I. O’Connor, Cascading effects of predator diversity and omnivory in a marine food web. Ecol. Lett. 8, 1048–1056 (2005).

[52] R. Cooke, W. Gearty, A. S. A. Chapman, J. Dunic, G. J. Edgar, J. S. Lefcheck, G. Rilov, C. R. McClain, R. D. Stuart-Smith, S. Kathleen Lyons, A. E. Bates, Anthropogenic disruptions to longstanding patterns of trophic-size structure in vertebrates. Nat. Ecol. Evol. 6, 684–692 (2022). 35449460 10.1038/s41559-022-01726-x

[53] J. A. Dunne, C. C. Labandeira, R. J. Williams, Highly resolved early Eocene food webs show development of modern trophic structure after the end-Cretaceous extinction. Proc. R. Soc. B. Biol. Sci. 281, 20133280 (2014). 10.1098/rspb.2013.3280 PMC3973268 24648225

[54] A. O. Achieng, G. B. Arhonditsis, N. Mandrak, C. Febria, B. Opaa, T. J. Coffey, F. O. Masese, K. Irvine, Z. M. Ajode, K. Obiero, J. E. Barasa, B. Kaunda-Arara, Monitoring biodiversity loss in rapidly changing Afrotropical ecosystems: an emerging imperative for governance and research. Philos. Trans. R. Sco. B. Biol. Sci. 378, 20220271 (2023). 10.1098/rstb.2022.0271 PMC10225856 37246384

[55] M. Chapman, B. R. Goldstein, C. J. Schell, J. S. Brashares, N. H. Carter, D. Ellis-Soto, H. O. Faxon, J. E. Goldstein, B. S. Halpern, J. Longdon, K. E. A. Norman, D. O’Rourke, C. Scoville, L. Xu, C. Boettiger, Biodiversity monitoring for a just planetary future. Science 383, 34–36 (2024). 38175872 10.1126/science.adh8874

[56] B. H. Daru, J. Rodriguez, Mass production of unvouchered records fails to represent global biodiversity patterns. Nat. Ecol. Evol. 7, 816–831 (2023). 37127769 10.1038/s41559-023-02047-3

[57] S. Kortsch, R. Frelat, L. Pecuchet, P. Olivier, I. Putnis, E. Bonsdorff, H. Ojaveer, I. Jurgensone, S. Strāķe, G. Rubene, Ē. Krūze, M. C. Nordström, Disentangling temporal food web dynamics facilitates understanding of ecosystem functioning. J. Anim. Ecol. 90, 1205–1216 (2021). 33608888 10.1111/1365-2656.13447

[58] S. H. M. Butchart, M. Walpole, B. Collen, A. van Strien, J. P. W. Scharlemann, R. E. A. Almond, J. E. M. Baillie, B. Bomhard, C. Brown, J. Bruno, K. E. Carpenter, G. M. Carr, J. Chanson, A. M. Chenery, J. Csirke, N. C. Davidson, F. Dentener, M. Foster, A. Galli, J. N. Galloway, P. Genovesi, R. D. Gregory, M. Hockings, V. Kapos, J.-F. Lamarque, F. Leverington, J. Loh, M. A. McGeoch, L. McRae, A. Minasyan, M. H. Morcillo, T. E. E. Oldfield, D. Pauly, S. Quader, C. Revenga, J. R. Sauer, B. Skolnik, D. Spear, D. Stanwell-Smith, S. N. Stuart, A. Symes, M. Tierney, T. D. Tyrrell, J.-C. Vié, R. Watson, Global biodiversity: Indicators of recent declines. Science 328, 1164–1168 (2010). 20430971 10.1126/science.1187512

[59] R. J. Williams, N. D. Martinez, Simple rules yield complex food webs. Nature 404, 180–183 (2000). 10724169 10.1038/35004572

[60] F. S. Scharf, F. Juanes, R. A. Rountree, Predator size-prey size relationships of marine fish predators: Interspecific variation and effects of ontogeny and body size on trophic-niche breadth. Mar. Ecol. Prog. Ser. 208, 229–248 (2000).

[61] F. He, C. Zarfl, V. Bremerich, J. N. W. David, Z. Hogan, G. Kalinkat, K. Tockner, S. C. Jähnig, The global decline of freshwater megafauna. Glob. Change Biol. 25, 3883–3892 (2019). 10.1111/gcb.14753 31393076

[62] N. Pacoureau, C. L. Rigby, P. M. Kyne, R. B. Sherley, H. Winker, J. K. Carlson, S. V. Fordham, R. Barreto, D. Fernando, M. P. Francis, R. W. Jabado, K. B. Herman, K.-M. Liu, A. D. Marshall, R. A. Pollom, E. V. Romanov, C. A. Simpfendorfer, J. S. Yin, H. K. Kindsvater, N. K. Dulvy, Half a century of global decline in oceanic sharks and rays. Nature 589, 567–571 (2021). 33505035 10.1038/s41586-020-03173-9

[63] C. D. Golden, J. Z. Koehn, A. Shepon, S. Passarelli, C. M. Free, D. F. Viana, H. Matthey, J. G. Eurich, J. A. Gephart, E. Fluet-Chouinard, E. A. Nyboer, A. J. Lynch, M. Kjellevold, S. Bromage, P. Charlebois, M. Barange, S. Vannuccini, L. Cao, K. M. Kleisner, E. B. Rimm, G. Danaei, C. DeSisto, H. Kelahan, K. J. Fiorella, D. C. Little, E. H. Allison, J. Fanzo, S. H. Thilsted, Aquatic foods to nourish nations. Nature 598, 315–320 (2021). 34526720 10.1038/s41586-021-03917-1 PMC10584661

[64] J. L. Blois, P. L. Zarnetske, M. C. Fitzpatrick, S. Finnegan, Climate change and the past, present, and future of biotic interactions. Science 341, 499–504 (2013). 23908227 10.1126/science.1237184

[65] M. Grenié, E. Berti, J. Carvajal-Quintero, G. M. L. Dädlow, A. Sagouis, M. Winter, Harmonizing taxon names in biodiversity data: A review of tools, databases and best practices. Methods Ecol. Evol. 14, 12–25 (2023).

[66] R. Froese, D. Pauly, FishBase (2023); https://www.fishbase.org/.

[67] C. Boettiger, D. T. Lang, P. C. Wainwright, rfishbase: Exploring, manipulating and visualizing FishBase data from R. J. Fish Biol. 81, 2030–2039 (2012). 23130696 10.1111/j.1095-8649.2012.03464.x

[68] R. Barnes, K. Sahr, G. Evenden, A. Johnson, F. Warmerdam, E. Rouault, L. Song, dggridR: Discrete Global Grids, version 3.0.0 (2023); https://cran.r-project.org/web/packages/dggridR/index.html.

[69] B. Lehner, G. Grill, Global river hydrography and network routing: Baseline data and new approaches to study the world’s large river systems. Hydrol. Process. 27, 2171–2186 (2013).

[70] A. Chao, L. Jost, Coverage-based rarefaction and extrapolation: Standardizing samples by completeness rather than size. Ecology 93, 2533–2547 (2012). 23431585 10.1890/11-1952.1

[71] N. J. Gotelli, R. K. Colwell, “Estimating species richness,” in *Biological Diversity: Frontiers in Measurement and Assessment* (Oxford Univ. Press, 2011), pp. 39–54.

[72] J. D. Carvajal-Quintero, Degradation of fish food webs in the anthropocene: Code and data used for Carvajal-Quintero *et al.* (2025); https://zenodo.org/records/15635349. 10.1126/sciadv.adu6540 PMC12915618 41706865

[73] J. H. Poelen, J. D. Simons, C. J. Mungall, Global biotic interactions: An open infrastructure to share and analyze species-interaction datasets. Ecol. Inform. 24, 148–159 (2014).

[74] S. R. Borstein, dietr: An R package for calculating fractional trophic levels from quantitative and qualitative diet data. Hydrobiologia 847, 4285–4294 (2020).

[75] I. Bartomeus, D. Gravel, J. M. Tylianakis, M. A. Aizen, I. A. Dickie, M. Bernard-Verdier, A common framework for identifying linkage rules across different types of interactions. Funct. Ecol. 30, 1894–1903 (2016).

[76] A. H. Hirzel, G. Le Lay, V. Helfer, C. Randin, A. Guisan, Evaluating the ability of habitat suitability models to predict species presences. Ecol. Model. 199, 142–152 (2006).

[77] C. Liu, G. Newell, M. White, J. Machunter, Improving the estimation of the Boyce index using statistical smoothing methods for evaluating species distribution models with presence-only data. Ecography 2025, e07218 (2025).

[78] C. Albouy, P. Archambault, W. Appeltans, M. B. Araújo, D. Beauchesne, K. Cazelles, A. R. Cirtwill, M.-J. Fortin, N. Galiana, S. J. Leroux, L. Pellissier, T. Poisot, D. B. Stouffer, S. A. Wood, D. Gravel, The marine fish food web is globally connected. Nat. Ecol. Evol. 3, 1153–1161 (2019). 31358950 10.1038/s41559-019-0950-y

[79] J. D. Yeakel, M. M. Pires, L. Rudolf, N. J. Dominy, P. L. Koch, P. R. Guimarães, T. Gross, Collapse of an ecological network in Ancient Egypt. Proc. Natl. Acad. Sci. U.S.A. 111, 14472–14477 (2014). 25201967 10.1073/pnas.1408471111 PMC4210013

[80] J. Fründ, K. S. McCann, N. M. Williams, Sampling bias is a challenge for quantifying specialization and network structure: Lessons from a quantitative niche model. Oikos 125, 502–513 (2016).

[81] R. M. Pringle, M. C. Hutchinson, Resolving food-web structure. Annu. Rev. Ecol. Evol. Syst. 51, 55–80 (2020).

[82] T. Poisot, E. Canard, D. Mouillot, N. Mouquet, D. Gravel, The dissimilarity of species interaction networks. Ecol. Lett. 15, 1353–1361 (2012). 22994257 10.1111/ele.12002

[83] I. Morales-Castilla, M. G. Matias, D. Gravel, M. B. Araújo, Inferring biotic interactions from proxies. Trends Ecol. Evol. 30, 347–356 (2015). 25922148 10.1016/j.tree.2015.03.014

[84] M. Pichler, V. Boreux, A.-M. Klein, M. Schleuning, F. Hartig, Machine learning algorithms to infer trait-matching and predict species interactions in ecological networks. Methods Ecol. Evol. 11, 281–293 (2020).

[85] M. Brooks, B. Bolker, K. Kristensen, M. Maechler, A. Magnusson, M. McGillycuddy, H. Skaug, A. Nielsen, C. Berg, K. van Bentham, N. Sadat, D. Lüdecke, R. Lenth, J. O’Brien, C. J. Geyer, M. Jagan, B. Wiernik, D. B. Stouffer, glmmTMB: Generalized Linear Mixed Models using Template Model Builder, version 1.1.8 (2023); https://cran.r-project.org/web/packages/glmmTMB/index.html.

[86] D. I. Bolnick, P. Amarasekare, M. S. Araújo, R. Bürger, J. M. Levine, M. Novak, V. H. W. Rudolf, S. J. Schreiber, M. C. Urban, D. A. Vasseur, Why intraspecific trait variation matters in community ecology. Trends Ecol. Evol. 26, 183–192 (2011). 21367482 10.1016/j.tree.2011.01.009 PMC3088364

[87] D. S. Rovinsky, A. R. Evans, D. G. Martin, J. W. Adams, Did the thylacine violate the costs of carnivory? Body mass and sexual dimorphism of an iconic Australian marsupial. Proc. R. Soc. B. Biol. Sci. 287, 20201537 (2020). 10.1098/rspb.2020.1537 PMC7482282 32811303

[88] R Core Team, R: A language and environment for statistical computing, R Foundation for Statistical Computing (2023); https://www.scirp.org/reference/referencespapers?referenceid=3582659.

[89] B. Efron, “Bootstrap methods: Another look at the jackknife,” in *Breakthroughs in Statistics: Methodology and Distribution*, S. Kotz, N. L. Johnson, Eds. (Springer, 1992), pp. 569–593; 10.1007/978-1-4612-4380-9_41.

