# Supplementary material for: Degradation of fish food webs in the Anthropocene
Source: Sci Adv. 2026 Feb 18;12(8):eadu6540. doi: 10.1126/sciadv.adu6540 (PMC12915618; doi:10.1126/sciadv.adu6540)
Supplement: Supplementary file 1 — Figs. S1 to S15 [file sciadv.adu6540_sm.pdf]

Science Advances
AAAS

Supplementary Materials for

Degradation of fish food webs in the Anthropocene

Juan D. Carvajal-Quintero *et al.*

Corresponding author: Juan D. Carvajal-Quintero, juan.carvajal@dal.ca

Sci. Adv. **12**, eadu6540 (2026)
DOI: 10.1126/sciadv.adu6540

This PDF file includes:

Figs. S1 to S15

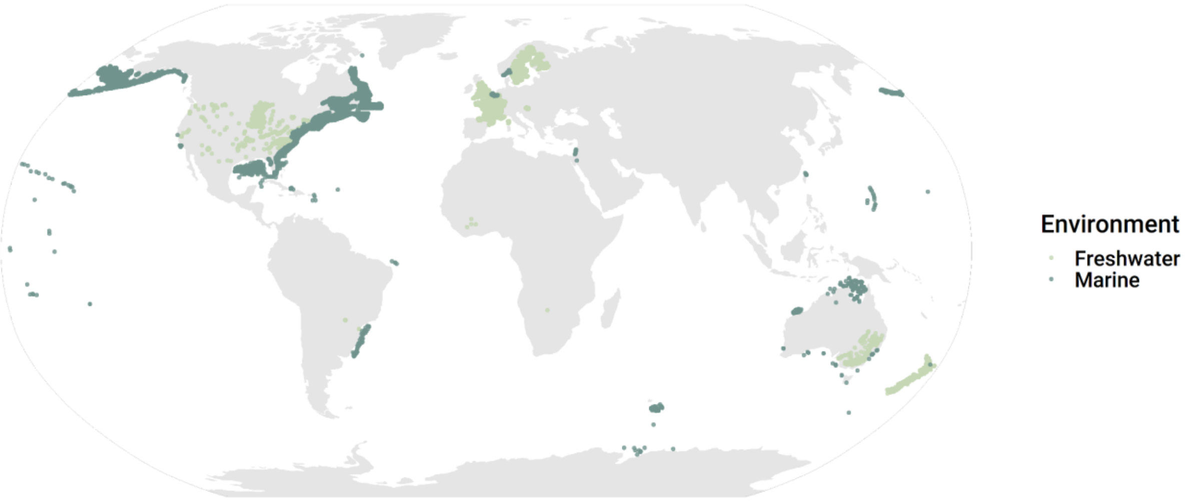

Fig. S1. Bivariate map depicting the spatial distribution of time series at the spatial unit level. Each dot denotes a fish-assemblage time series with a reconstructed food web. Dark green dots denote fish assemblages in marine environments, while light green denotes freshwater assemblages.

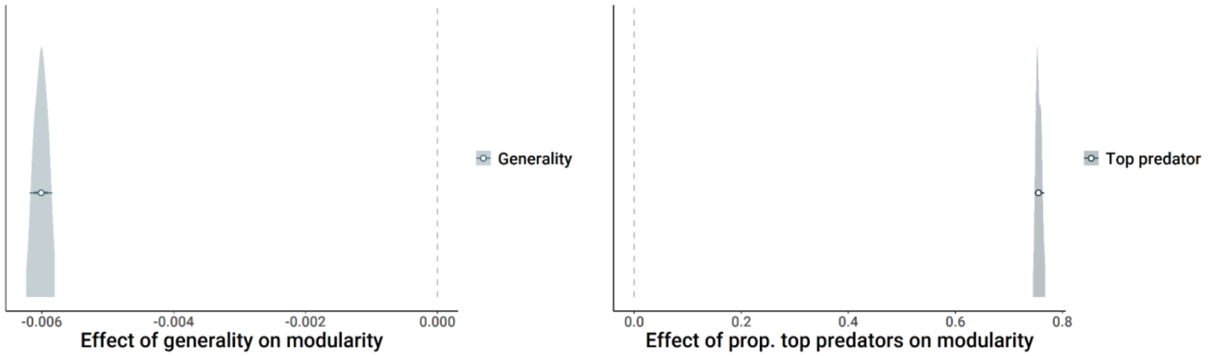

Fig. S2. Effect of generality and proportion of top predators on modularity. The density plots display the distribution of effect size of the generality and the proportion of top predator species within the food webs in network modularity across the 100 iterations of the rarefied time series. The horizontal error bars denote the 50% and 95% confidence intervals (CIs) of the mean estimates (depicted by white circles). Both generality and the proportion of top predators exhibited consistently positive effects on modularity, with low variability in their effect sizes.

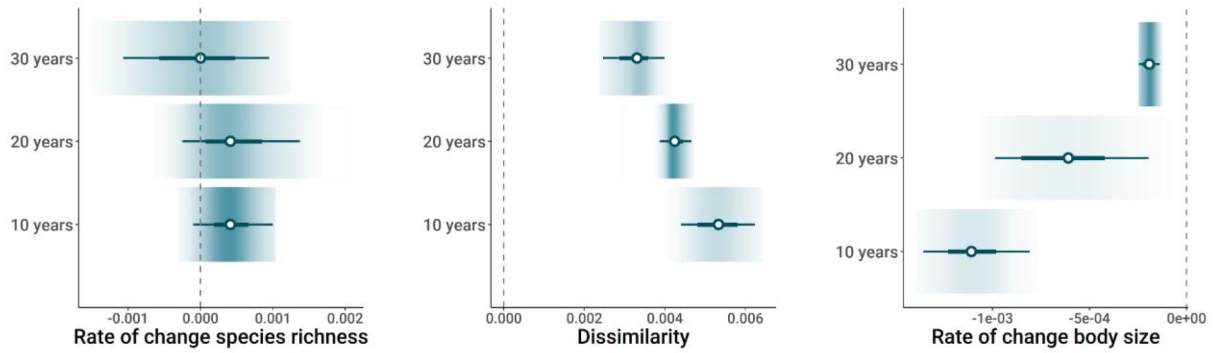

Fig. S3. Changes in assemblage metrics in time series spanning at least 10, 20 and 30 years. The gradient plots display the distribution of slopes for changes in species richness, dissimilarity in species composition, and body size across the 100 iterations of the rarefied time series. Darker colors correspond to higher densities. The horizontal error bars denote the 50% and 95% confidence intervals (CIs) of the mean estimates (depicted by white circles).

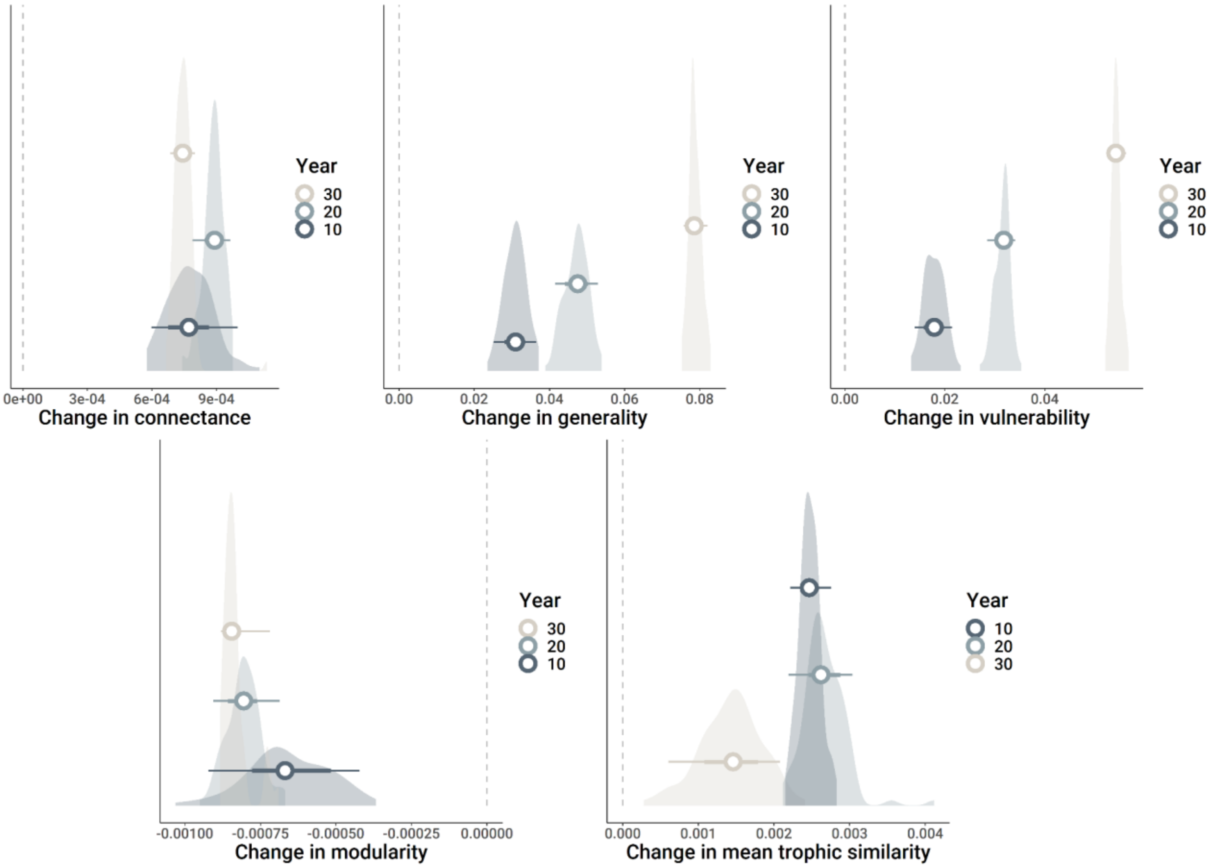

Fig. S4. Changes in food web topology metrics and trophic similarity in time series spanning at least 10, 20 and 30 years. The density plots display the distribution of slopes for changes in connectance, generality, prey vulnerability (predation pressure), modularity, and trophic similarity across the 100 iterations of the rarefied time series. The horizontal error bars denote the 50% and 95% confidence intervals (CIs) of the mean estimates (depicted by white circles).

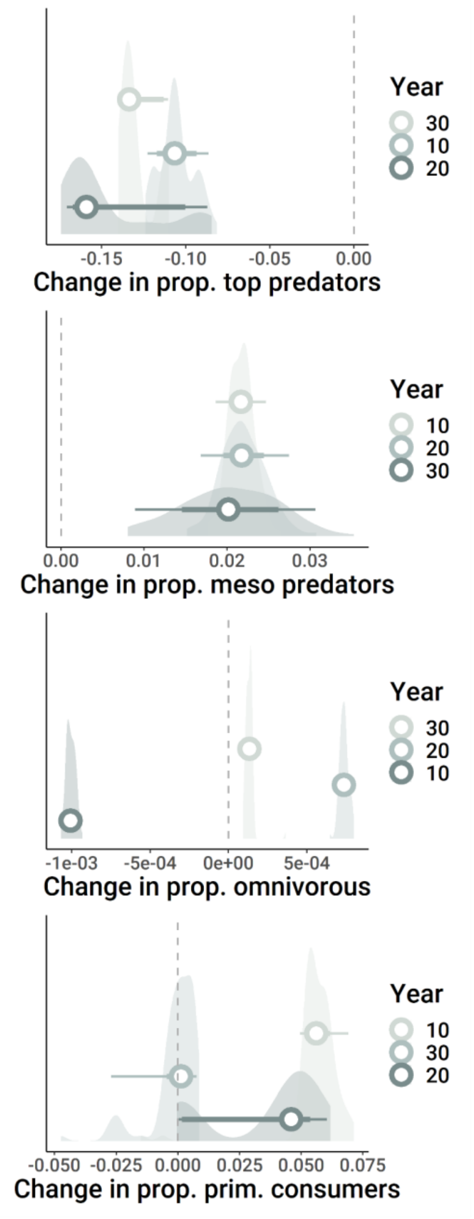

Fig. S5. Changes in proportion of trophic groups in time series spanning at least 10, 20 and 30 years. The density plots display the distribution of slopes for changes in proportion within the assemblages of top predators, mesopredators, omnivorous, and primary consumers across the 100 iterations of the rarefied time series. The horizontal error bars denote the 50% and 95% confidence intervals (CIs) of the mean estimates (depicted by white circles).

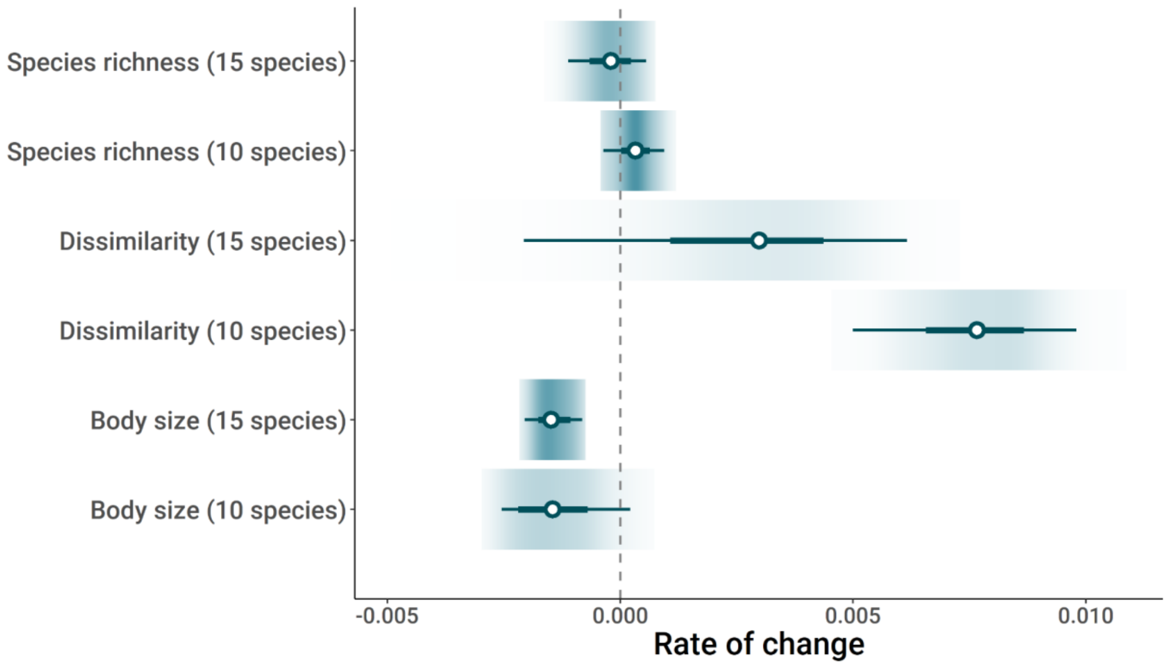

Fig. S6. Changes in assemblage metrics in time series with at least 10 and 15 species. The gradient plots display the distribution of slopes for changes in species richness, dissimilarity in species composition, and body size across the 100 iterations of the rarefied time series. Darker colors correspond to higher densities. The horizontal error bars denote the 50% and 95% confidence intervals (CIs) of the mean estimates (depicted by white circles).

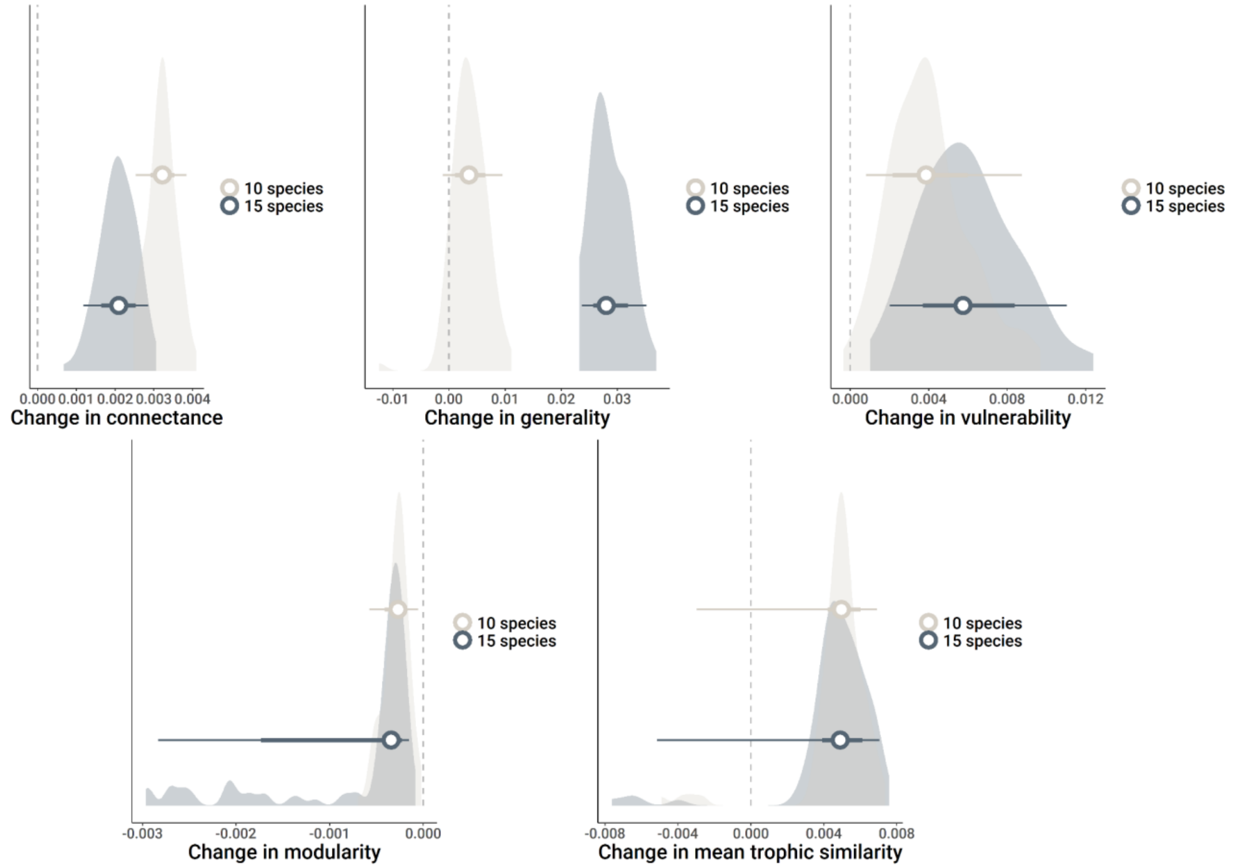

Fig. S7. Changes in food web topology metrics and trophic similarity in time series with at least 10 and 15 species. The density plots display the distribution of slopes for changes in connectance, generality, prey vulnerability (predation pressure), modularity, and trophic similarity across the 100 iterations of the rarefied time series. The horizontal error bars denote the 50% and 95% confidence intervals (CIs) of the mean estimates (depicted by white circles).

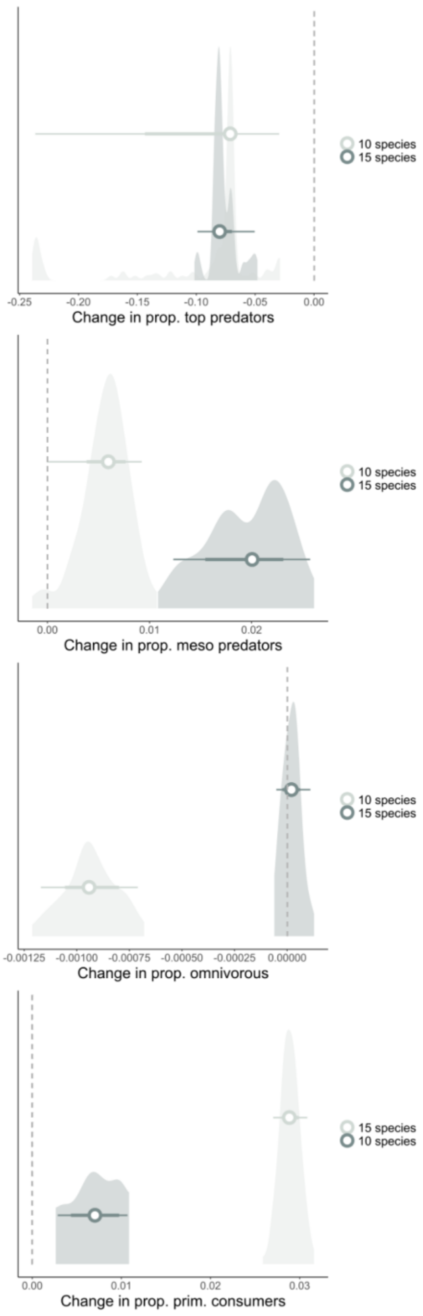

Fig. S8. Changes in the proportion of trophic groups in time series with at least 10 and 15 species. The density plots display the distribution of slopes for changes in proportion within the assemblages of top predators, mesopredators, omnivorous, and primary consumers across the 100 iterations of the rarefied time series. The horizontal error bars denote the 50% and 95% confidence intervals (CIs) of the mean estimates (depicted by white circles).

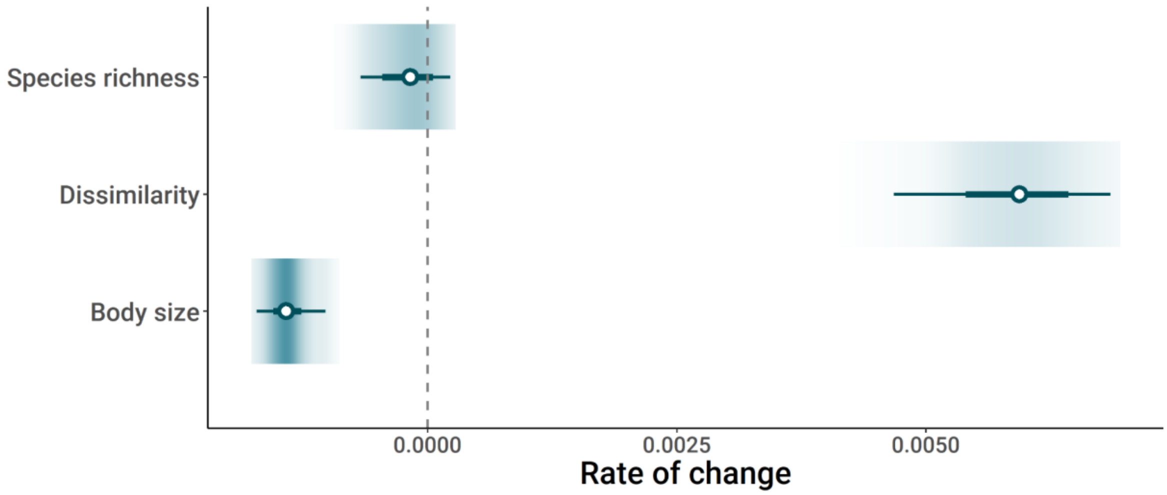

Fig. S9. Changes in assemblage metrics in time series with a sampling coverage of 100%. The gradient plots display the distribution of slopes for changes in species richness, dissimilarity in species composition, and body size across the 100 iterations of the rarefied time series. Darker colors correspond to higher densities. The horizontal error bars denote the 50% and 95% confidence intervals (CIs) of the mean estimates (depicted by white circles).

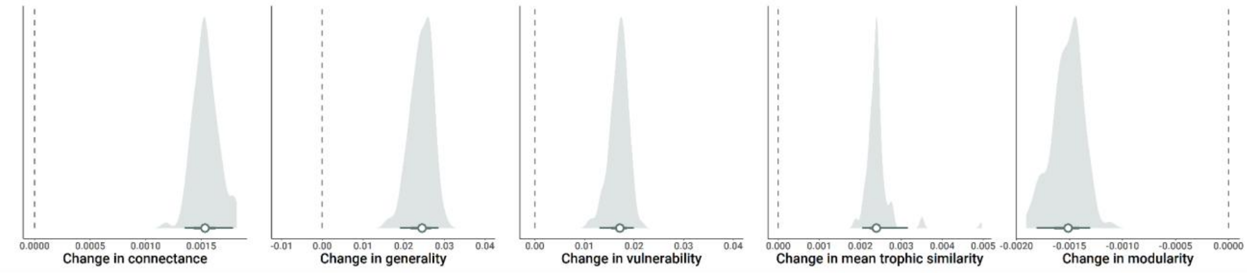

Fig. S10. Changes in food web topology metrics and trophic similarity in time series with a sampling coverage of 100%. The density plots display the distribution of slopes for changes in connectance, generality, prey vulnerability (predation pressure), mean trophic similarity, and modularity across the 100 iterations of the rarefied time series. The horizontal error bars denote the 50% and 95% confidence intervals (CIs) of the mean estimates (depicted by white circles).

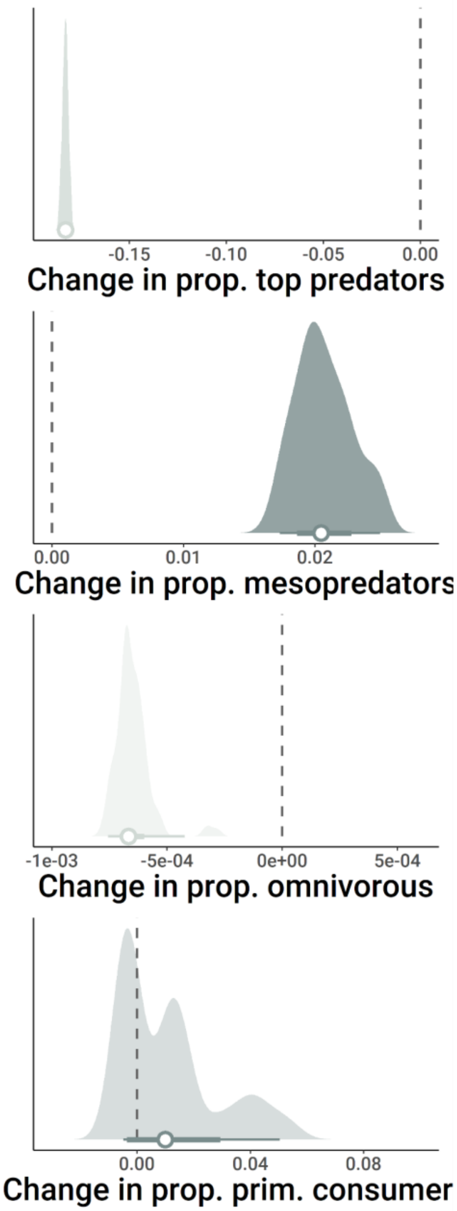

Fig. S11. Changes in the proportion of trophic groups in time series with a sampling coverage of 100%. The density plots display the distribution of slopes for changes in proportion within the assemblages of top predators, mesopredators, omnivorous, and primary consumers across the 100 iterations of the rarefied time series. The horizontal error bars denote the 50% and 95% confidence intervals (CIs) of the mean estimates (depicted by white circles).

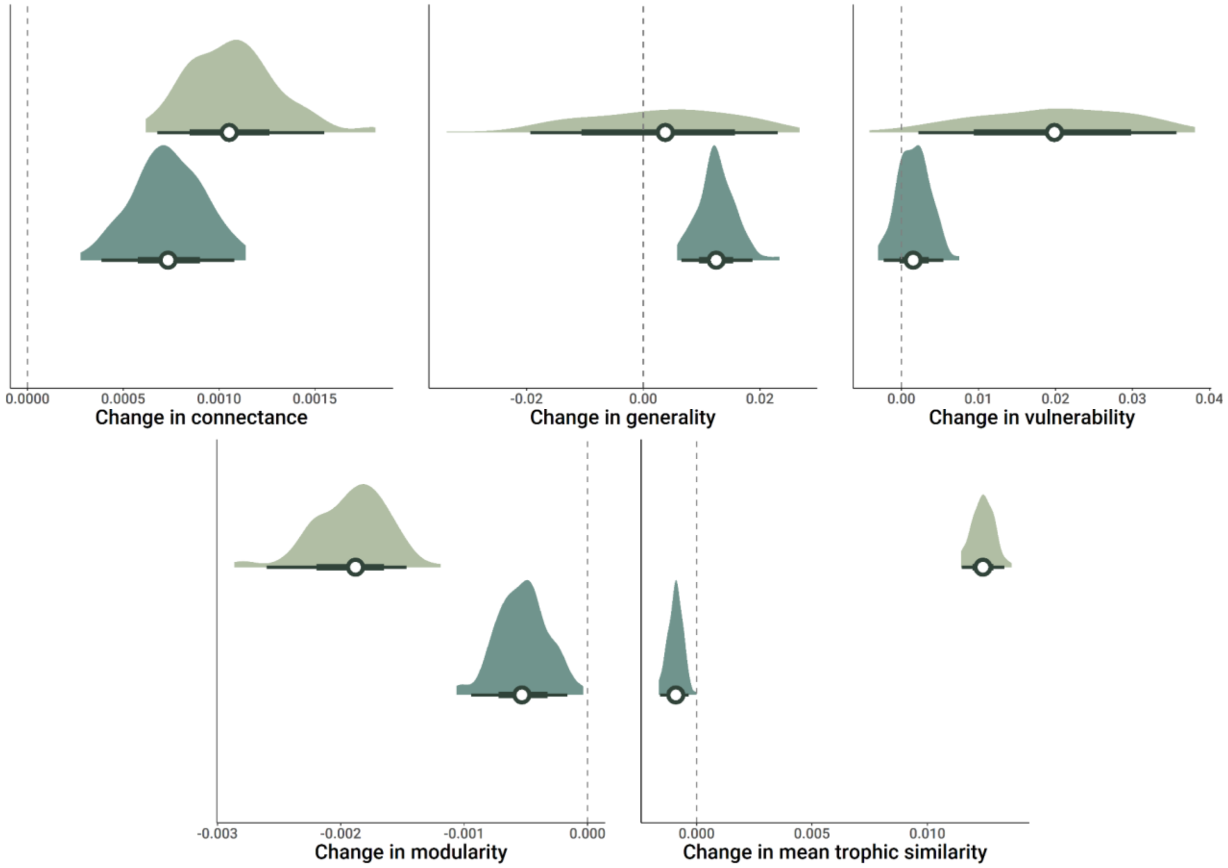

Fig. S12. Changes in food web topology metrics and trophic similarity in marine (dark green) and freshwater environments (light green). The density plots display the distribution of slopes for changes in connectance, generality, vulnerability (predation pressure), modularity, and mean trophic similarity across the 100 iterations of the rarefied time series. The horizontal error bars denote the 50% and 95% confidence intervals (CIs) of the mean estimates (depicted by white circles).

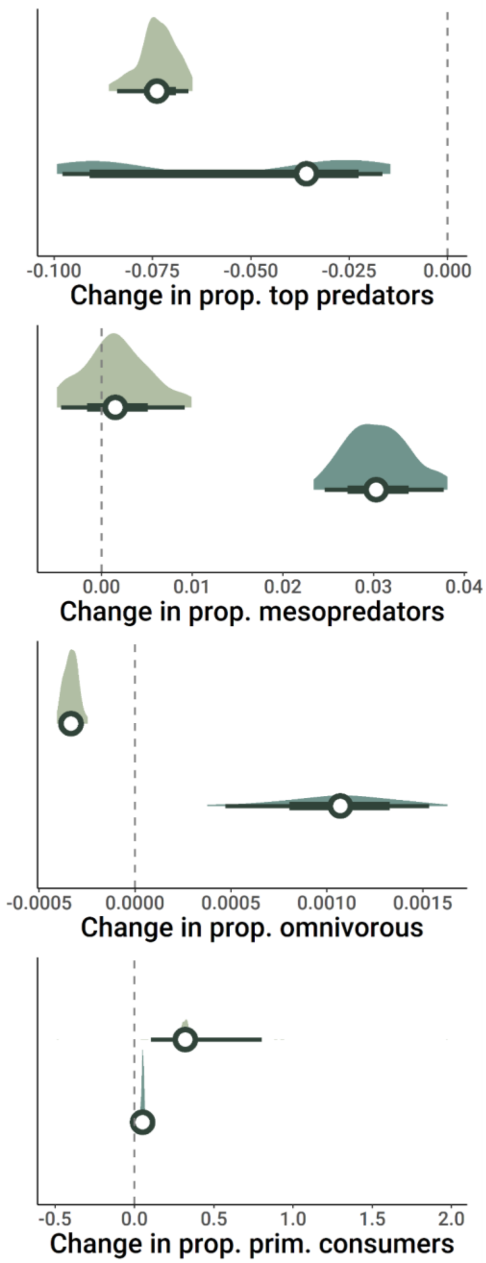

Fig. S13. Changes in the proportion of trophic groups between marine (dark green) and freshwater environments (light green). The density plots display the distribution of slopes for changes in proportion within the assemblages of top predators, mesopredators, omnivorous, and primary consumers across the 100 iterations of the rarefied time series. The horizontal error bars denote the 50% and 95% confidence intervals (CIs) of the mean estimates (depicted by white circles).

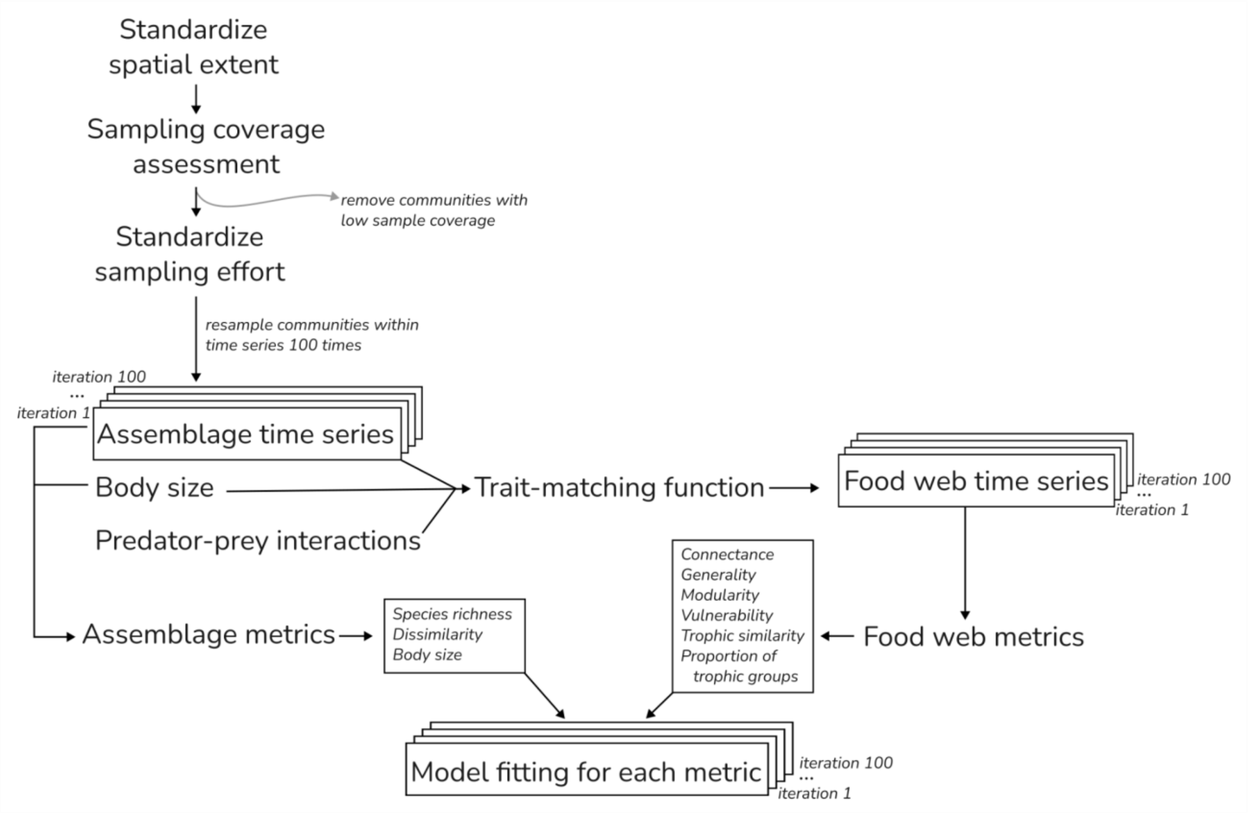

Fig. S14. Steps of data pre-processing and analyses. The overlapped boxes illustrate the iterations of sample-based rarefaction conducted to standardize the number of samples per year within each time series.

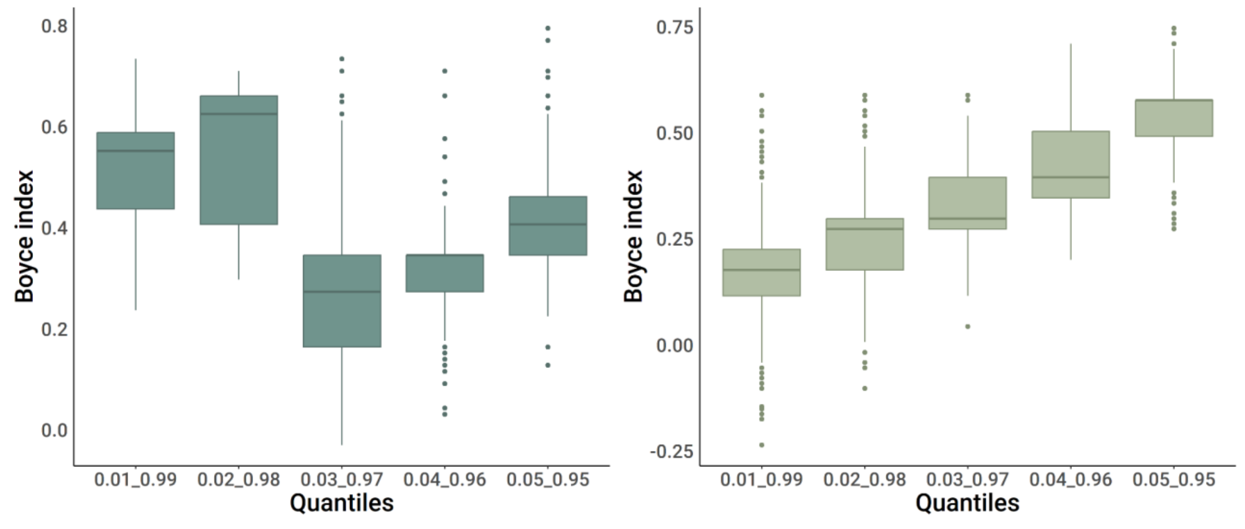

Fig. S15. Assessment of niche model calibration process. The niche model was fine-tuned using an independent dataset of predator-prey interactions, employing various predation windows (quantiles) for fish species found in marine (dark green) and freshwater environments (light green). Models exhibiting superior performance, as determined by the Boyce index (Marine fish 0.05-0.95, mean Boyce index = 0.56, s.d. = 0.09, n = 999; Freshwater fish 0.03-0.97, mean Boyce index = 0.53, s.d. = 0.08, n = 999), were chosen to reconstruct trophic interactions within the fish assemblages.
